# Supplementary figures and images for: Unraveling the genetic links between stature and disease in East Asians: A multi-biobank genetic correlation and risk prediction study
Source: PLoS Genet. 2026 Mar 13;22(3):e1012030. doi: 10.1371/journal.pgen.1012030 (PMC13001983; doi:10.1371/journal.pgen.1012030)

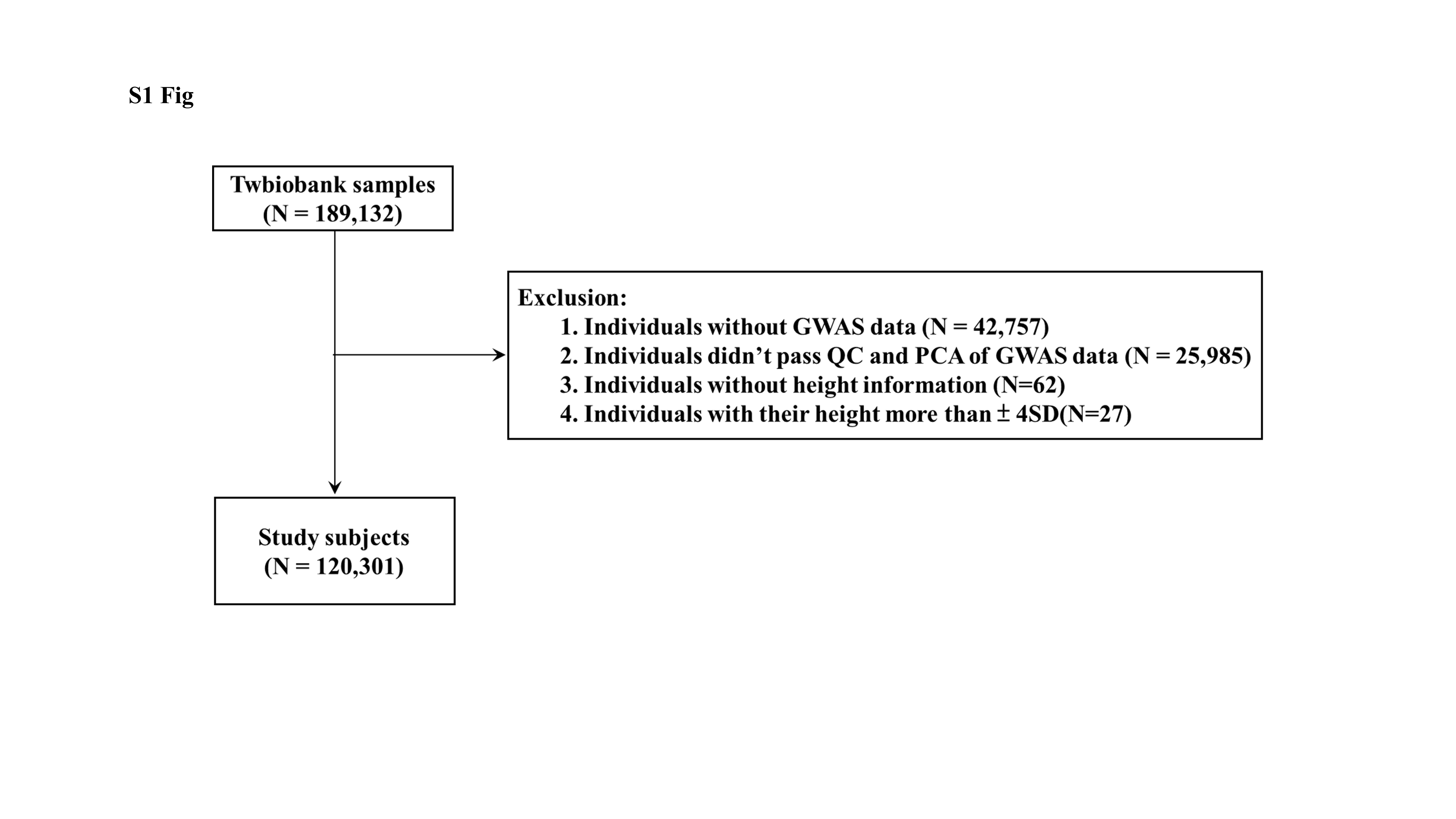

Supplement: S1 Fig — Starting from 189,132 TWB participants, individuals were excluded based on the following criteria: missing GWAS data (N = 42,757), failure to pass GWAS quality control (QC) and principal component analysis (PCA) (N = 25,985), missing height information (N = 62), and extreme height values beyond ±4 standard deviations (SD) from the mean (N = 27). (TIF) [file pgen.1012030.s001.tif]

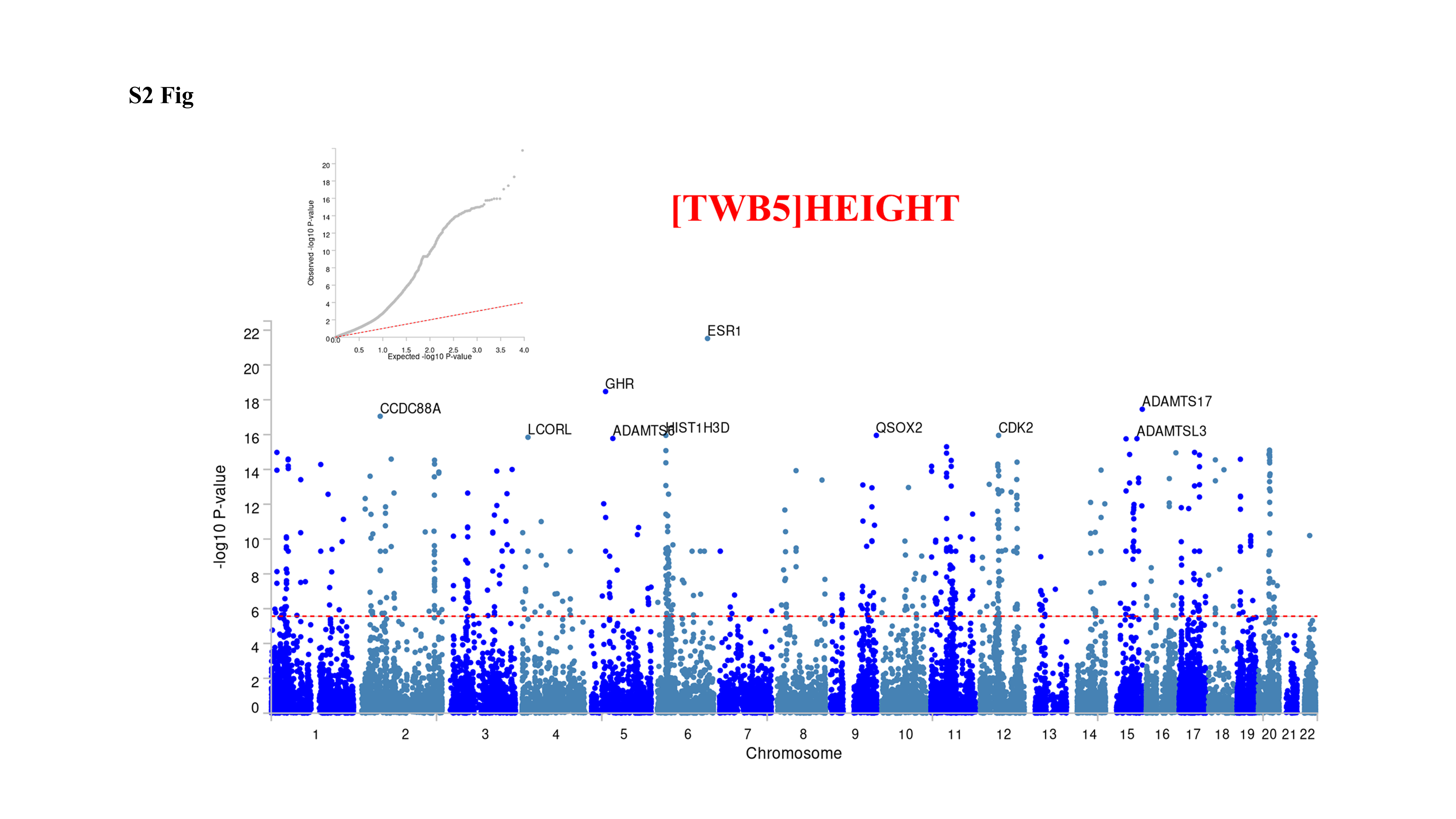

Supplement: S2 Fig — (TIF) [file pgen.1012030.s002.tif]

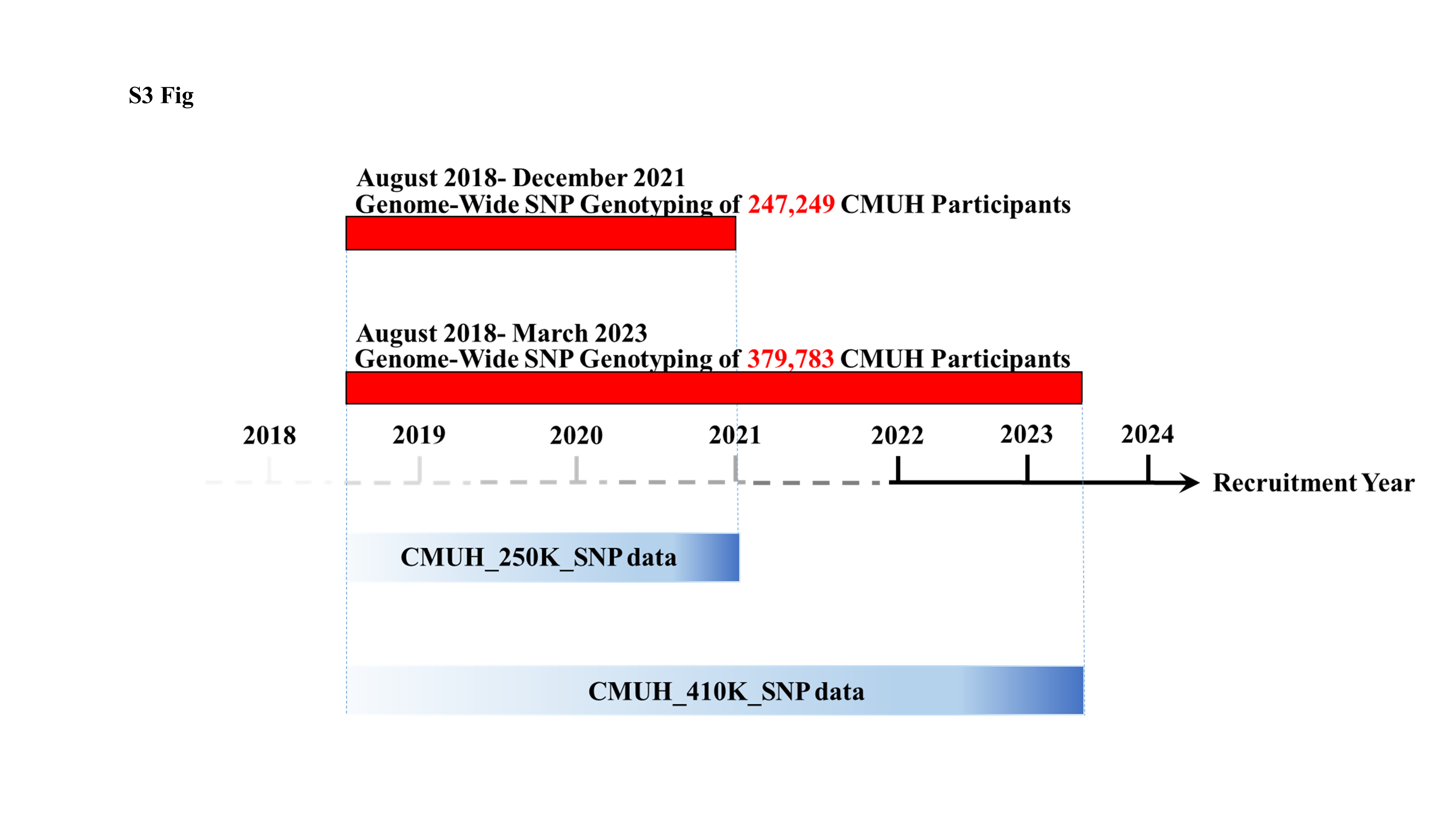

Supplement: S3 Fig — The first genotyping phase (August 2018–December 2021) included 247,249 participants, and the second phase (August 2018–March 2023) included 379,783 participants. The CMUH_250K_SNP dataset corresponded to data generated during the first phase, while the CMUH_410K_SNP dataset corresponded to the expanded data collection. Abbreviations: CMUH, China Medical University Hospital; SNP, single-nucleotide polymorphism. (TIF) [file pgen.1012030.s003.tif]

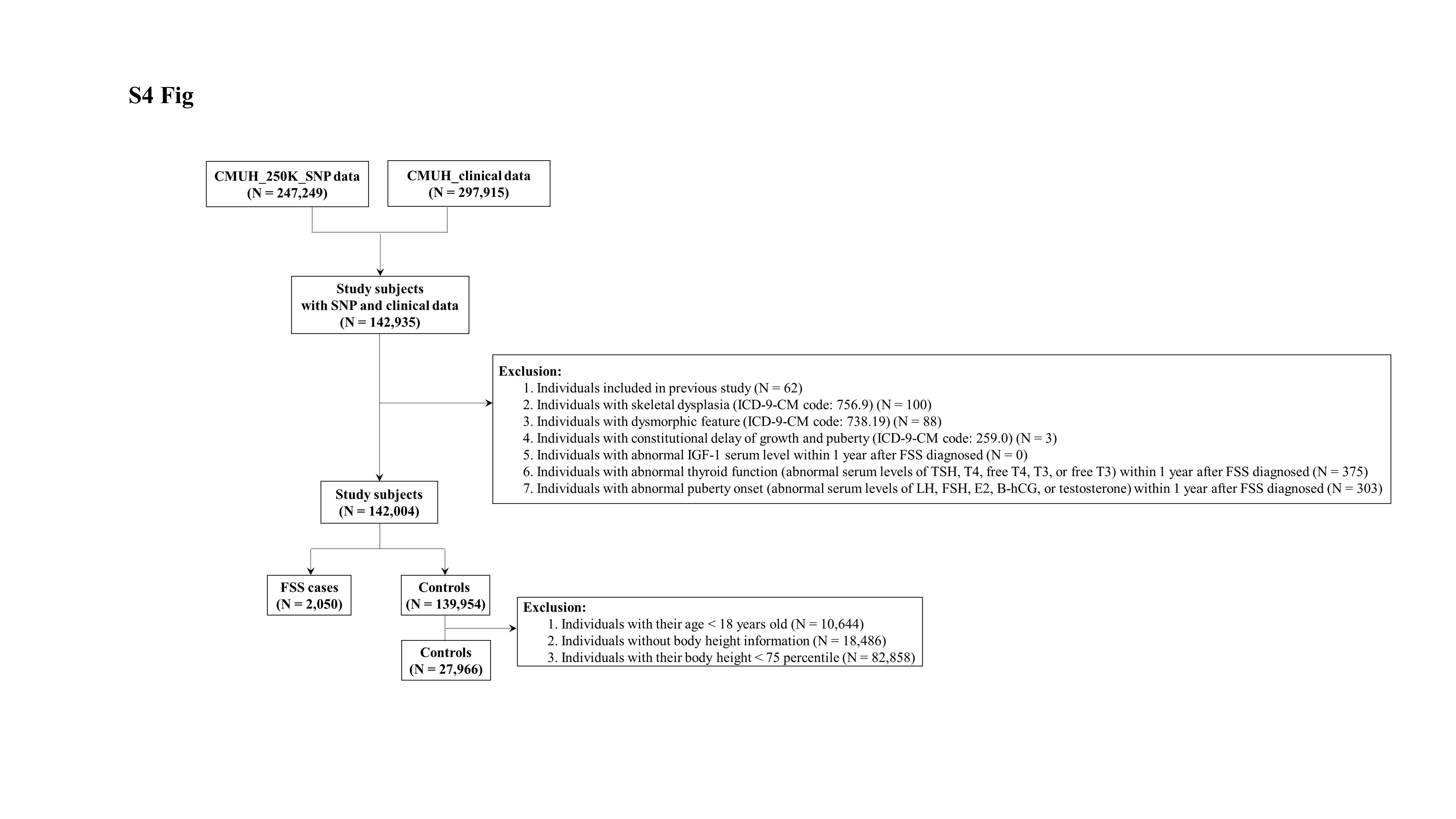

Supplement: S4 Fig — The CMUH_250K_SNP dataset comprised 247,249 participants with genotyping data, and the CMUH_clinical dataset included 297,915 participants with electronic health records. A total of 142,935 participants had both SNP and clinical data, from which 2,050 familial short stature (FSS) cases and 27,966 controls were identified for genome-wide association analysis. (TIF) [file pgen.1012030.s004.tif]

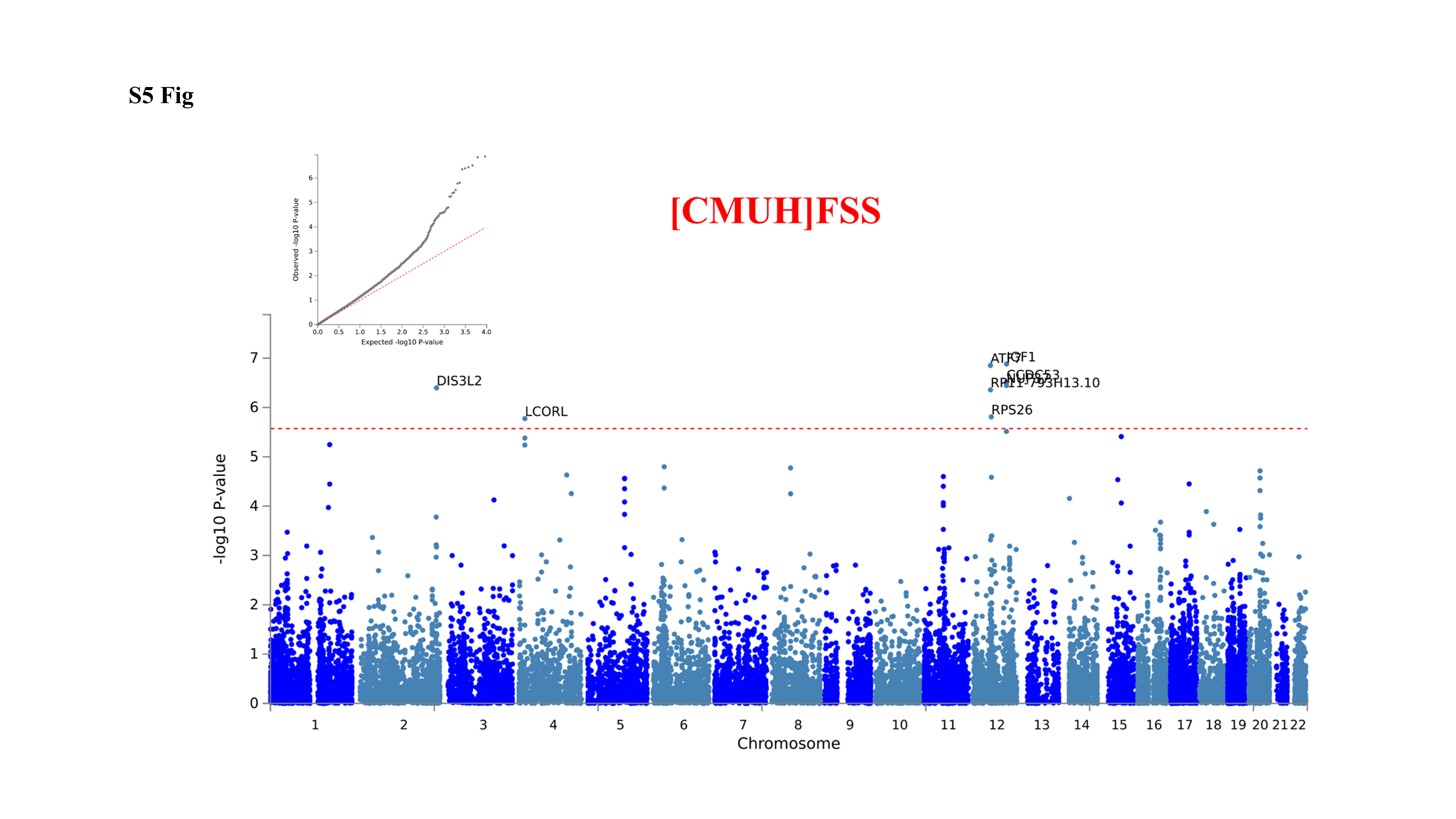

Supplement: S5 Fig — (TIF) [file pgen.1012030.s005.tif]

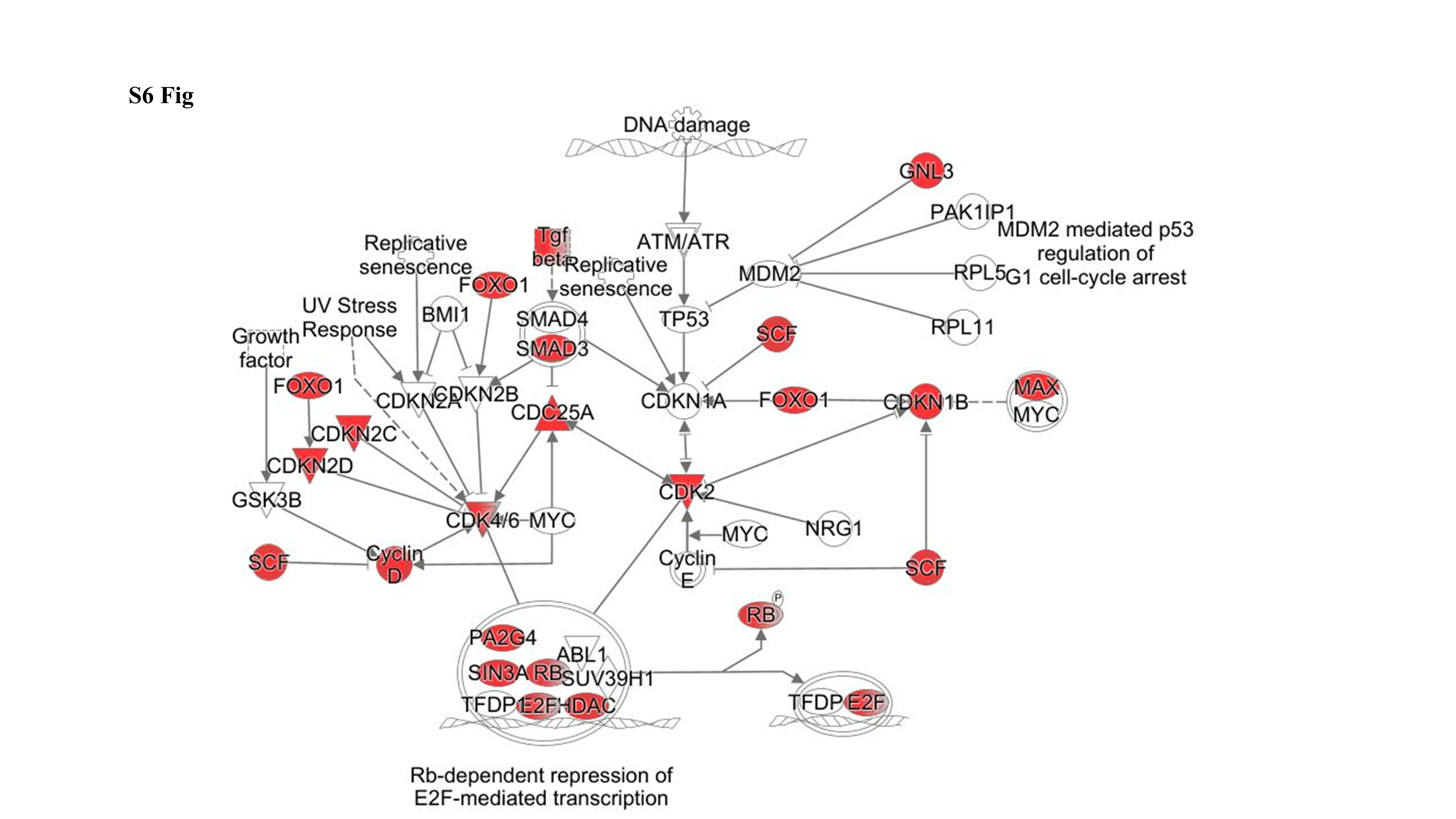

Supplement: S6 Fig — Abbreviations: IPA, Ingenuity Pathway Analysis; TWB5, Taiwan Biobank (version 5); Korean Genome and Epidemiology Study; CMUH, China Medical University Hospital; FSS, familial short stature. (TIF) [file pgen.1012030.s006.tif]

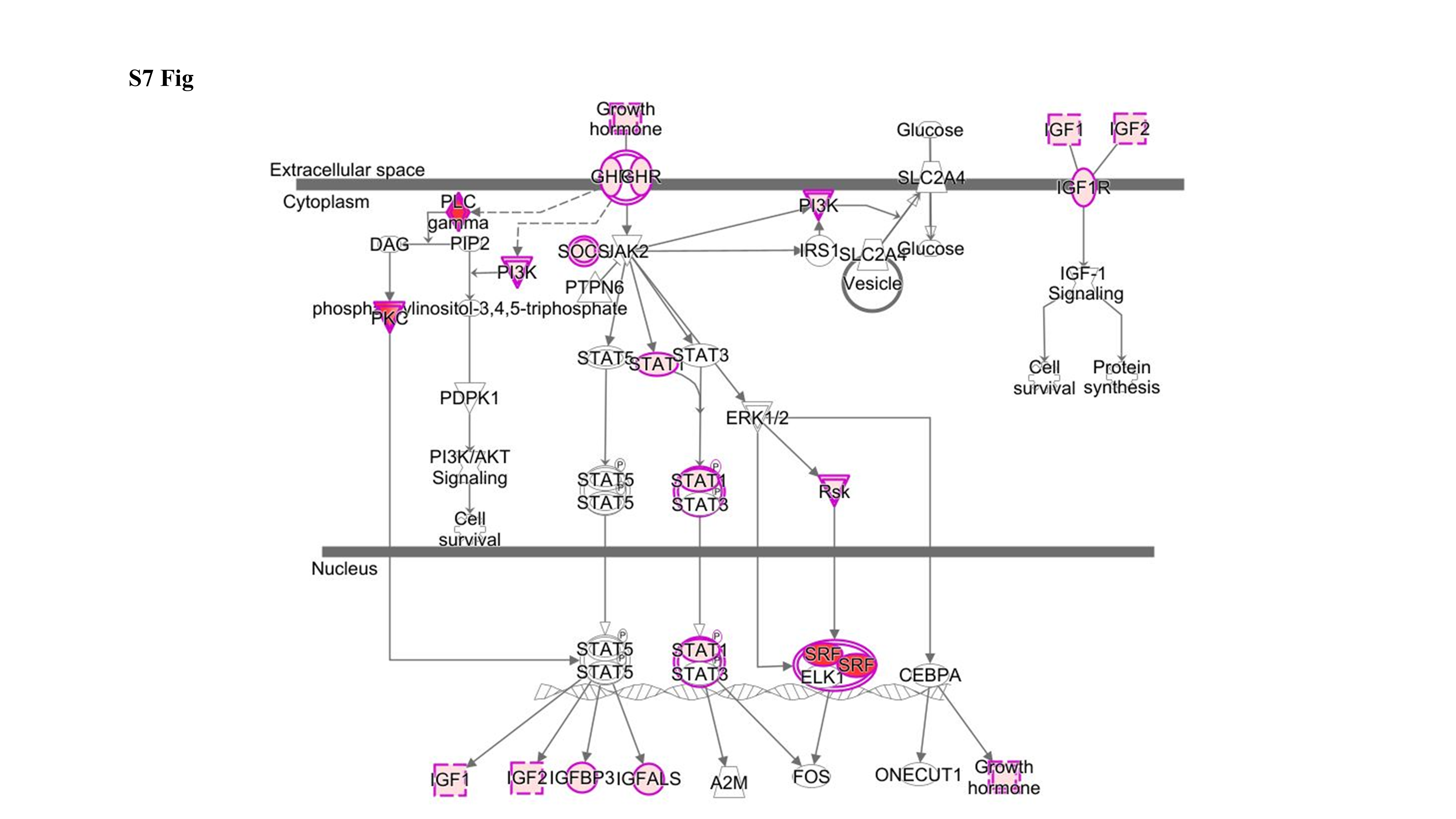

Supplement: S7 Fig — Abbreviations: IPA, Ingenuity Pathway Analysis; TWB5, Taiwan Biobank (version 5); Korean Genome and Epidemiology Study; CMUH, China Medical University Hospital; FSS, familial short stature. (TIF) [file pgen.1012030.s007.tif]

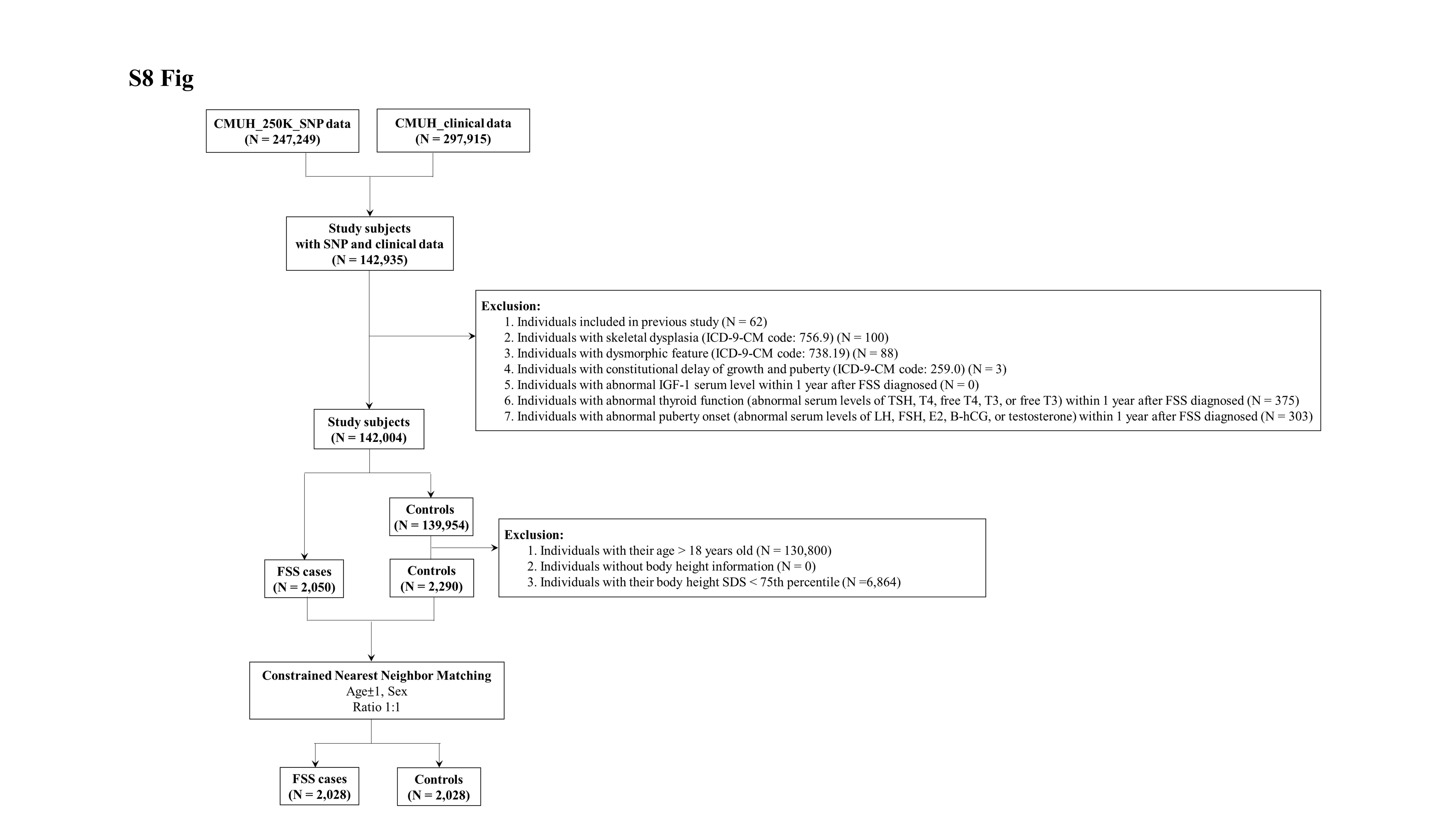

Supplement: S8 Fig — Data integration of the CMUH_250K_SNP (N = 247,249) and CMUH_clinical (N = 297,915) datasets identified 142,935 subjects with overlapping records. Following the exclusion of individuals with prior study involvement or specific diagnoses (e.g., skeletal dysplasia, dysmorphic features, and abnormal thyroid/puberty function), 142,004 subjects remained. To generate a robust control set, individuals aged > 18 years or with height SDS < 75th percentile were removed. Constrained nearest neighbor matching (1:1 ratio, age ± 1 year, sex-matched) was performed to balance the final dataset, resulting in 2,028 FSS cases and 2,028 matched controls. (TIF) [file pgen.1012030.s008.tif]

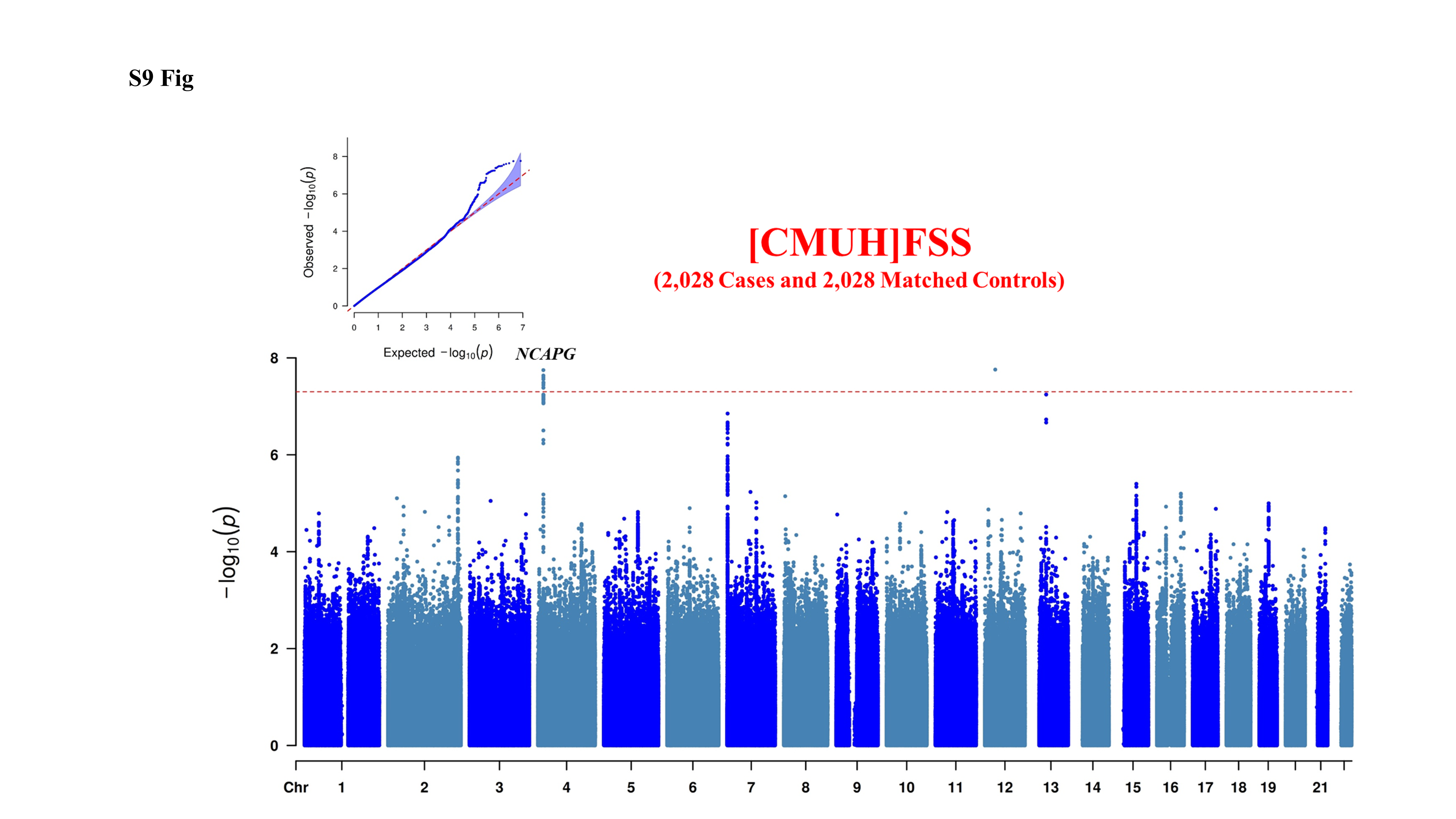

Supplement: S9 Fig — Manhattan and Quantile-Quantile (QQ) plots summarized the sensitivity analysis comparing 2,028 FSS cases with 2,028 controls from the CMUH cohort. Controls were selected via constrained nearest neighbor matching (1:1 ratio, age ± 1 year, sex-matched) to balance the dataset. The red dashed line indicated the genome-wide significance threshold (P < 5 × 10^-8^), with the NCAPG locus labeled. The inset displayed the QQ plot of observed versus expected –log10 (P) values. (TIF) [file pgen.1012030.s009.tif]

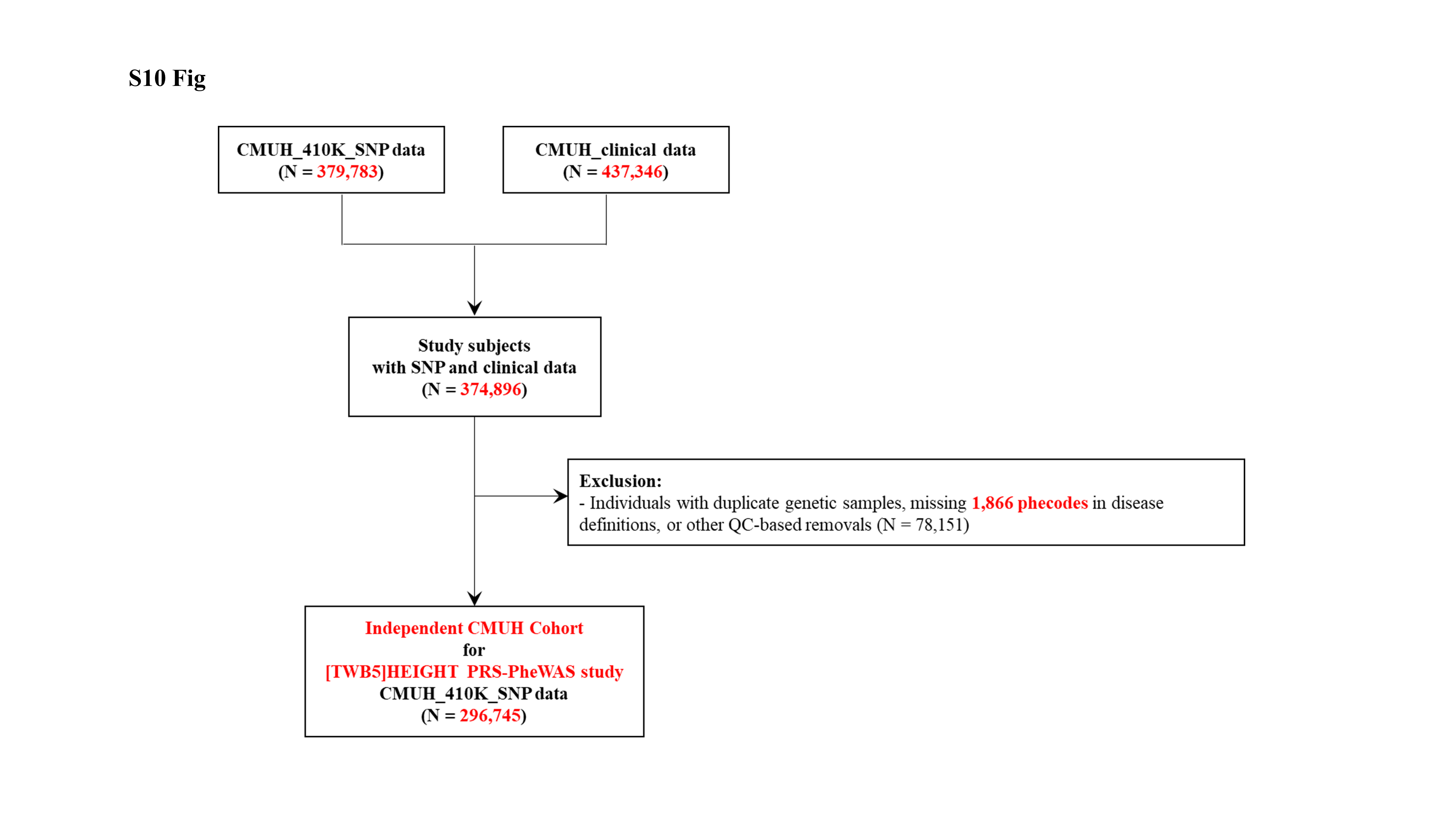

Supplement: S10 Fig — Starting with 379,783 individuals from the CMUH_410K_SNP dataset and 437,346 from the CMUH clinical database, 374,896 participants with both SNP and clinical data were identified. After exclusions for duplicate genetic samples, missing 1,866 phecodes in disease definitions, or other QC-based removals (N = 78,151), a total of 296,745 individuals were retained for the final analysis cohort. Abbreviations: CMUH, China Medical University Hospital; TWB5, Taiwan Biobank (version 5); PRS, polygenic risk score; PheWAS, phenome-wide association study; SNP, single-nucleotide polymorphism; QC, quality control. (TIF) [file pgen.1012030.s010.tif]

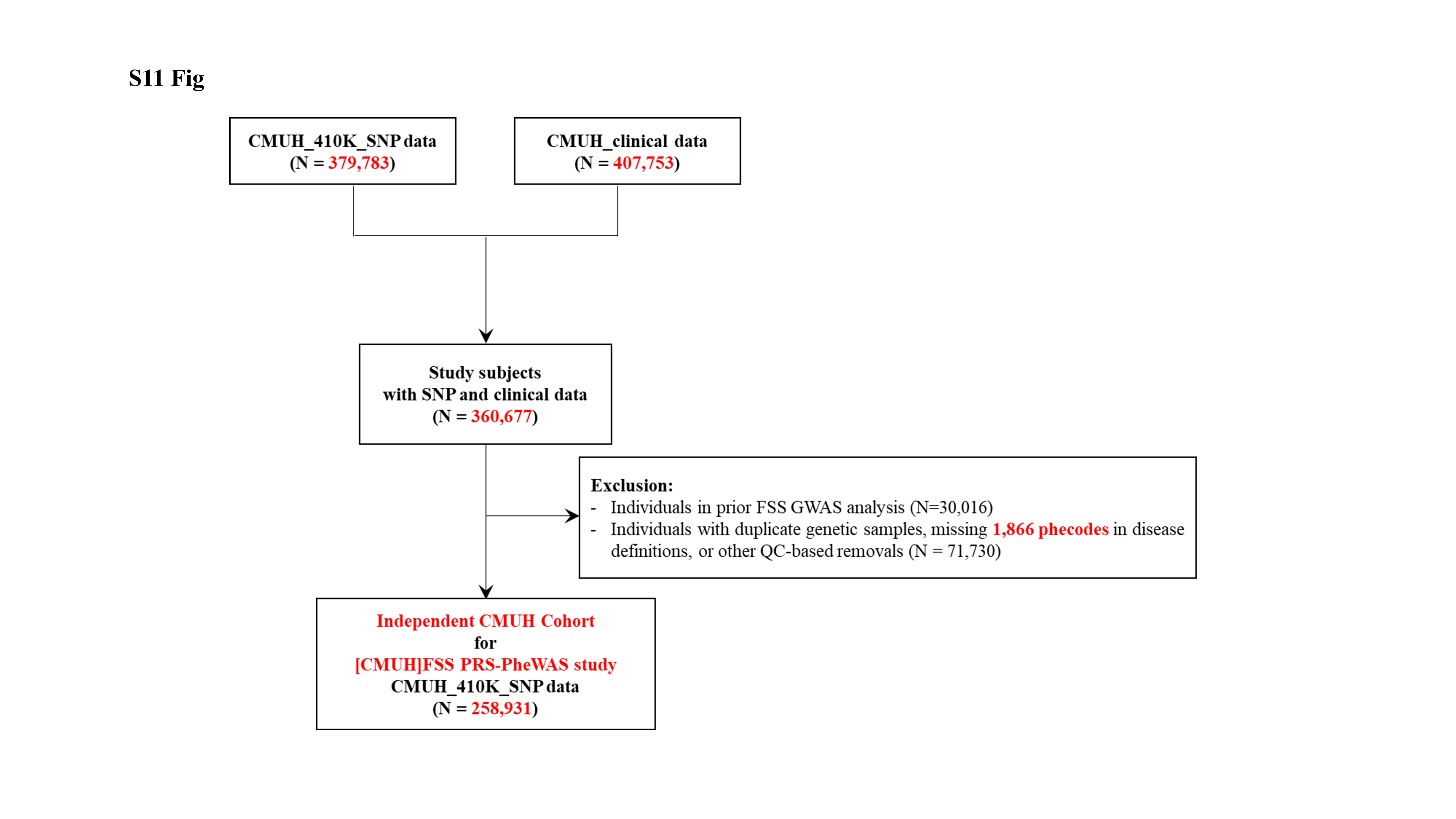

Supplement: S11 Fig — From 379,783 individuals in the CMUH_410K_SNP dataset and 407,753 in the CMUH clinical database, 360,677 with both SNP and clinical data were identified. After exclusions for prior FSS GWAS participation, duplicate samples, missing 1,866 phecodes, and other QC-based removals, 258,931 participants remained for analysis. Abbreviations: CMUH, China Medical University Hospital; FSS, familial short stature; PRS, polygenic risk score; PheWAS, phenome-wide association study; SNP, single-nucleotide polymorphism; GWAS, genome-wide association study; QC, quality control. (TIF) [file pgen.1012030.s011.tif]

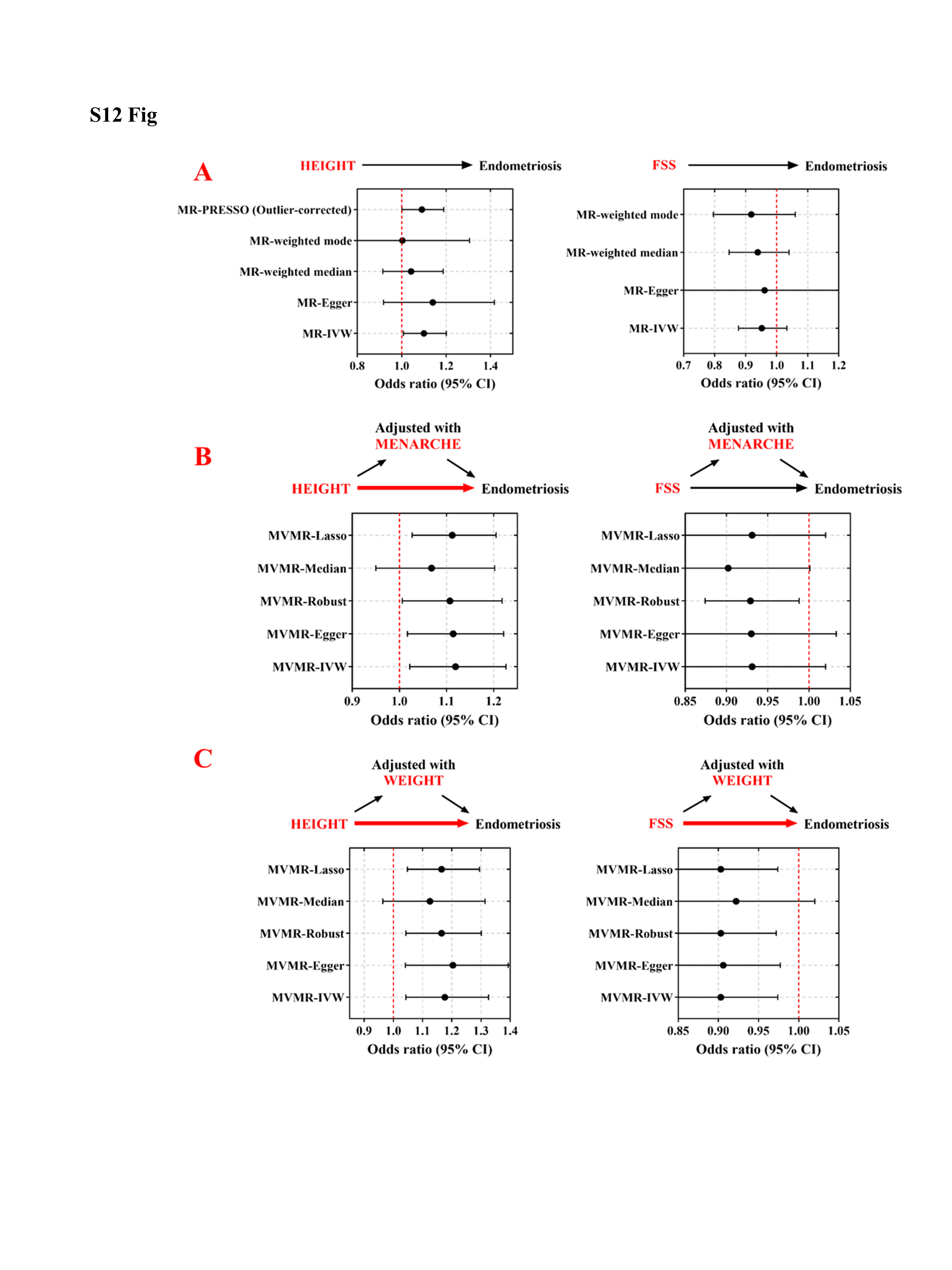

Supplement: S12 Fig — A. Causal effect of stature on endometriosis in East Asians using two-sample Mendelian randomization (MR). Left: Effect of [TWB5] HEIGHT on [CMUH] Endometriosis; Right: Effect of [CMUH] familial short stature (FSS) on [CMUH] endometriosis. B. Causal effect of stature on endometriosis in East Asians using multivariate MR adjusted for age at menarche. Left: Effect of [TWB5] HEIGHT on [CMUH] Endometriosis; Right: Effect of [CMUH] familial short stature (FSS) on [CMUH] endometriosis. [TWB5] MENARCHE was included as the covariate in MVMR. C. Causal effect of stature on endometriosis in East Asians using multivariate MR adjusted for body weight. Left: Effect of [TWB5] HEIGHT on [CMUH] Endometriosis; Right: Effect of [CMUH] familial short stature (FSS) on [CMUH] endometriosis. [TWB5] WEIGHT was included as the covariate in MVMR. Abbreviations: TWB5, Taiwan Biobank (version 5); HEIGHT, body height; CMUH, China Medical University Hospital; FSS, familial short stature; MR, Mendelian randomization; MVMR, multivariate MR; WEIGHT, body weight; MENARCHE, age at menarche. (TIF) [file pgen.1012030.s012.tif]

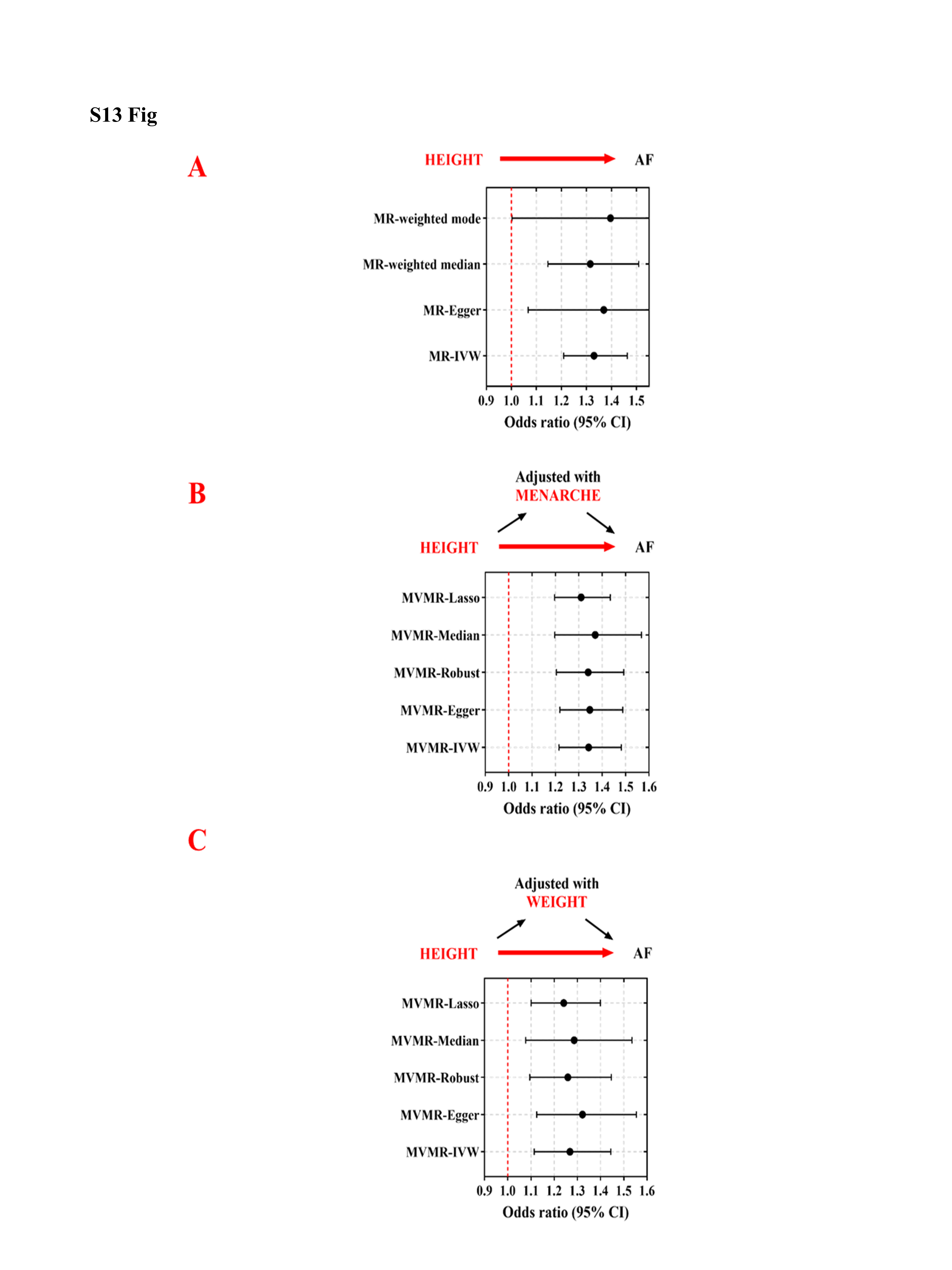

Supplement: S13 Fig — A. Causal effect of body height on atrial flutter/fibrillation in East Asians using two-sample Mendelian randomization (MR). Effect of [TWB5] HEIGHT on [BBJ]AF. B. Causal effect of body height on atrial flutter/fibrillation in East Asians using multivariate MR adjusted for age at menarche. Effect of [TWB5] HEIGHT on [BBJ]AF. [TWB5] MENARCHE was included as the covariate in MVMR. C. Causal effect of body height on atrial flutter/fibrillation in East Asians using multivariate MR adjusted for body weight. Effect of [TWB5] HEIGHT on [BBJ]AF. [TWB5] WEIGHT was included as the covariate in MVMR. Abbreviations: TWB5, Taiwan Biobank (version 5); BBJ, Biobank Japan; HEIGHT, body height; AF, atrial flutter/fibrillation; MR, Mendelian randomization; MVMR, multivariate MR; WEIGHT, body weight; MENARCHE, age at menarche. (TIF) [file pgen.1012030.s013.tif]

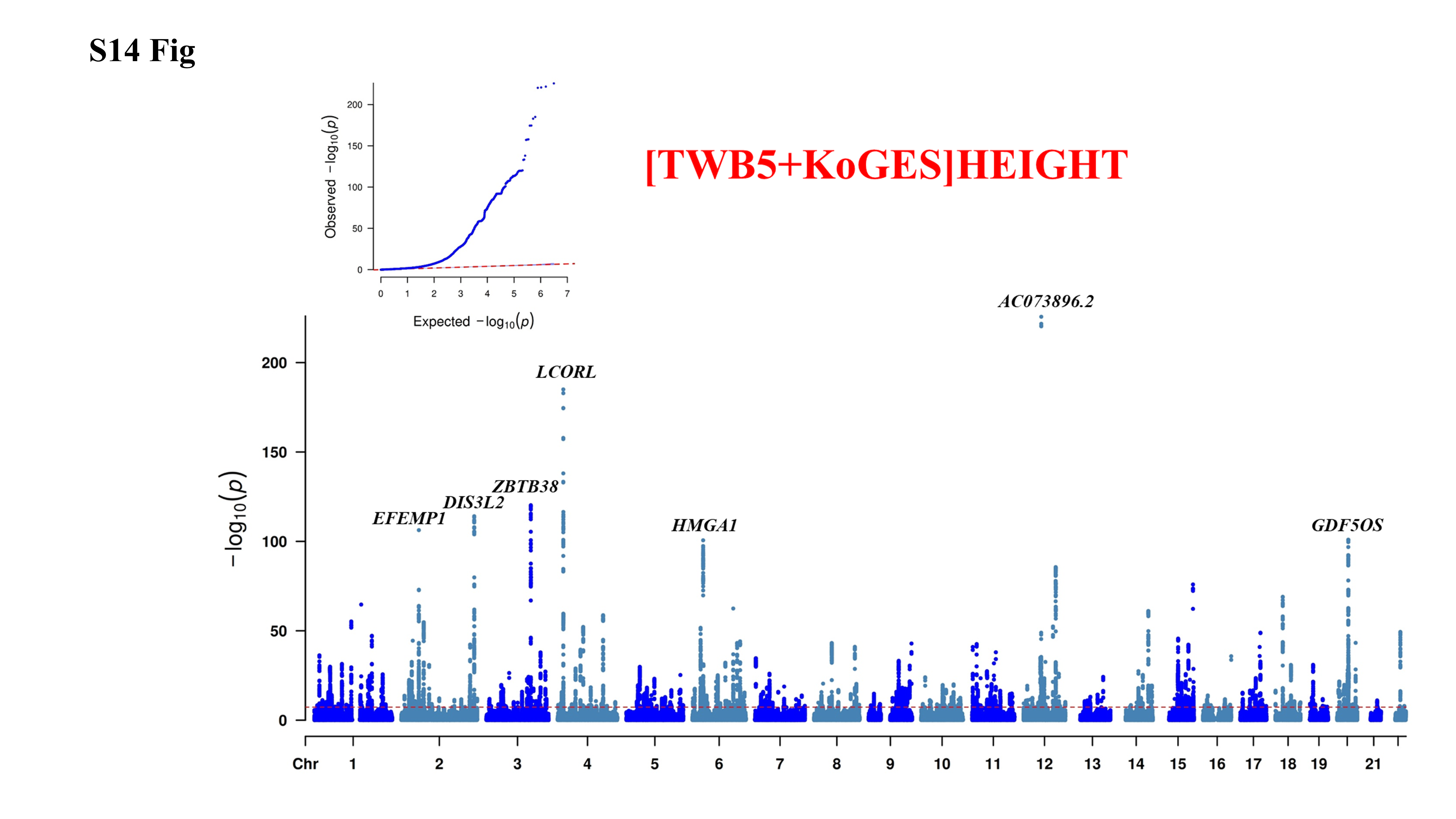

Supplement: S14 Fig — (TIF) [file pgen.1012030.s014.tif]

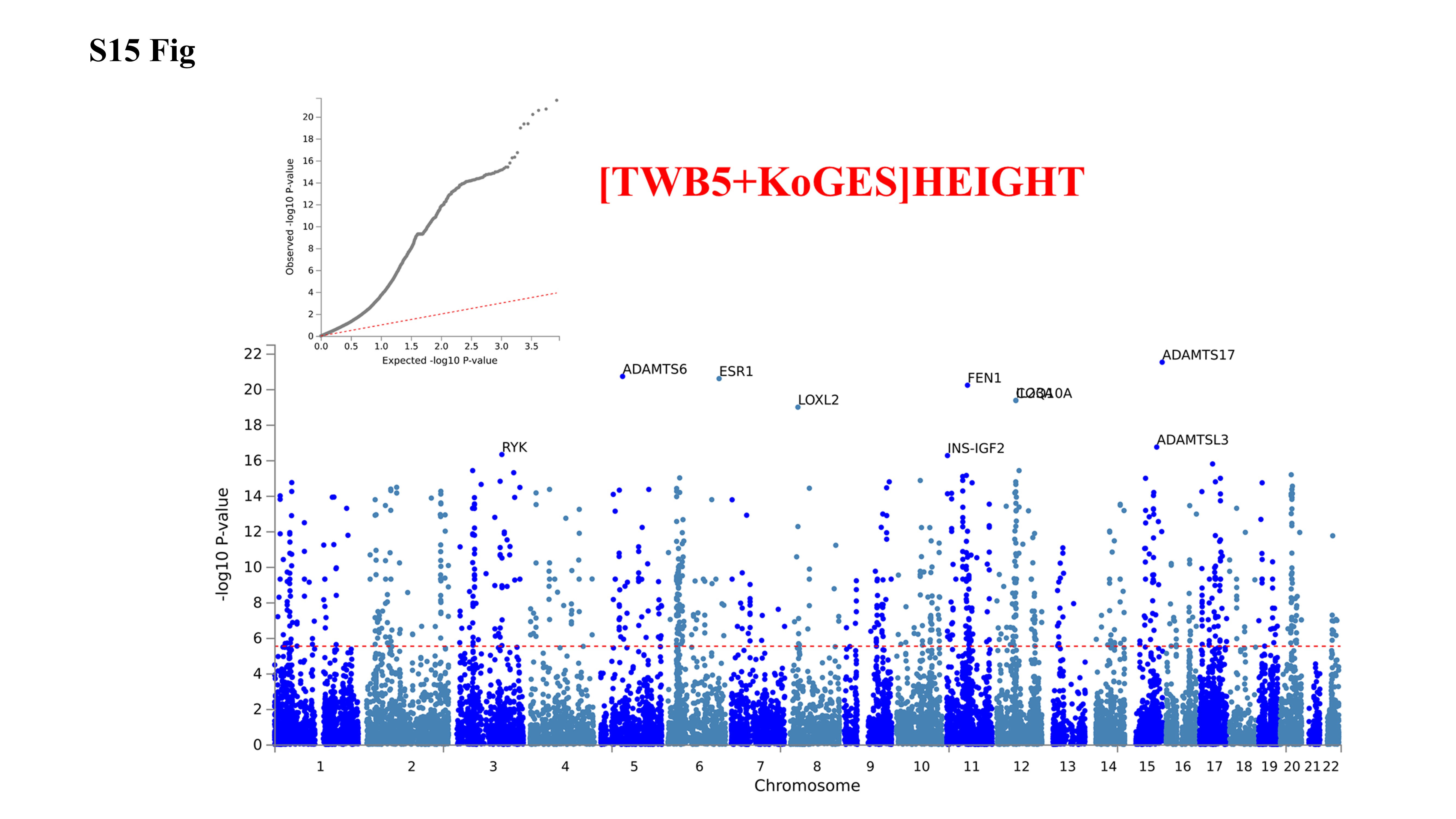

Supplement: S15 Fig — (TIF) [file pgen.1012030.s015.tif]

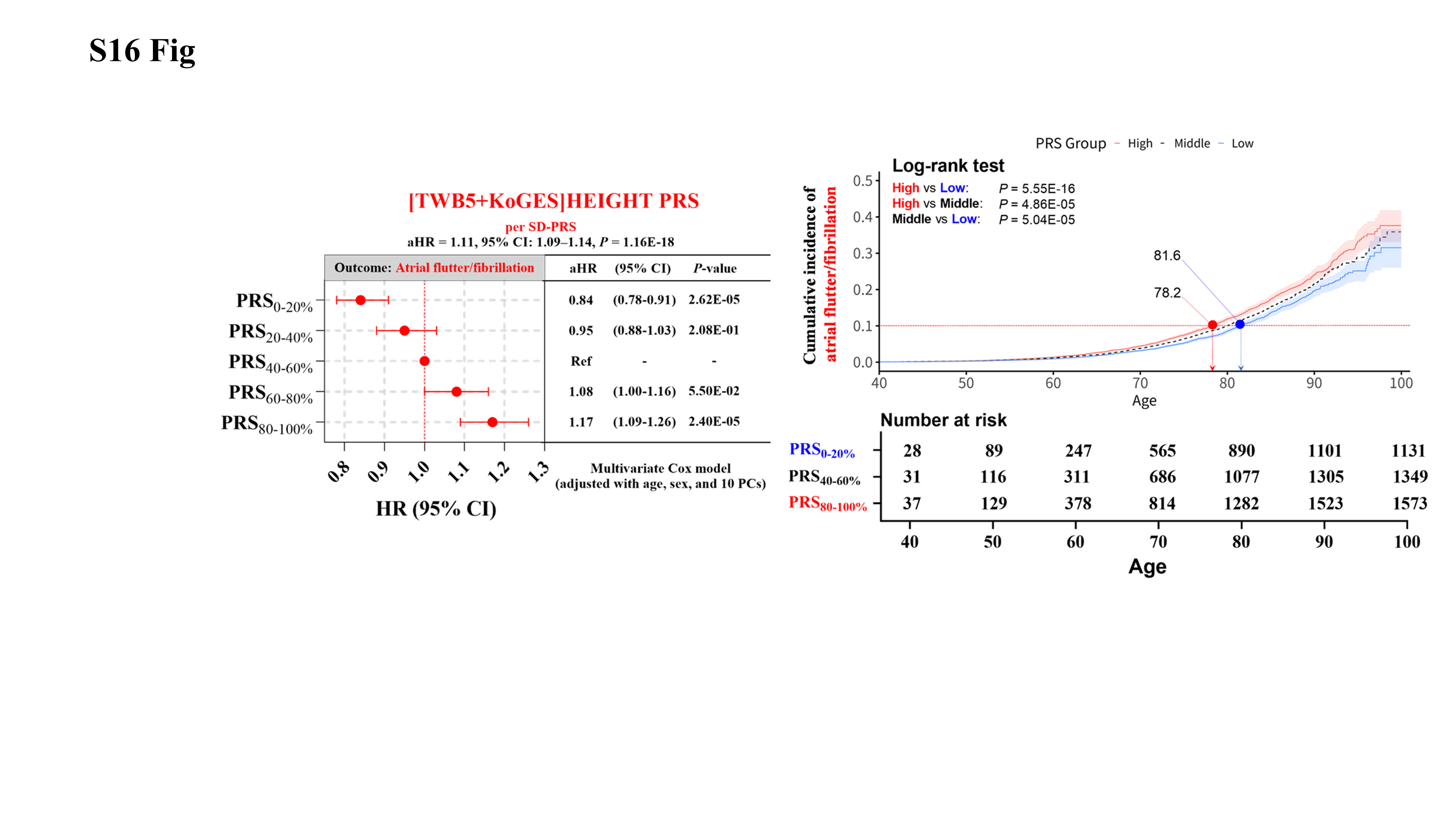

Supplement: S16 Fig — Left panel: Multivariate Cox model results for atrial flutter/fibrillation risk across height meta-analysis PRS quintiles, presenting hazard ratios (HRs) and 95% confidence intervals (CIs) relative to the reference group (PRS40–60%), adjusted for age, sex, and 10 principal components. Right panel: Kaplan–Meier survival curves illustrating the cumulative incidence of atrial flutter/fibrillation among individuals with low (PRS0–20%), middle (PRS40–60%), and high (PRS80–100%) genetic risk. P-values are based on log-rank tests comparing differences across PRS groups. The dashed line denotes the age at which each PRS group reaches a cumulative incidence of 10%. Numbers displayed below the curves represent participants at risk at each time point for the corresponding groups. Abbreviations: AF, atrial flutter/fibrillation; CI, confidence interval; HR, hazard ratio; PCs, principal components; PRS, polygenic risk score. (TIF) [file pgen.1012030.s016.tif]
