# Supplementary material for: Unraveling the genetic links between stature and disease in East Asians: A multi-biobank genetic correlation and risk prediction study
Source: PLoS Genet. 2026 Mar 13;22(3):e1012030. doi: 10.1371/journal.pgen.1012030 (PMC13001983; doi:10.1371/journal.pgen.1012030)
Supplement: Attachment — Submitted filename: Response_to_PLOS genetics_10102025.docx [file pgen.1012030.s060.docx]

PLOS Genetics

MS ID: PGENETICS-D-25-00676

MS TITLE: Unraveling the genetic links between stature and disease in East Asians: A multi-biobank genetic correlation and risk prediction study

Dear Professors Themistocles L. Assimes, Anne O'Donnell-Luria, Aimée Dudley, and Anne Goriely:

Thank you for your e-mail dated July 23, informing us that you and the reviewer have suggested a revision for our manuscript. We greatly appreciate this opportunity, and we have made changes with red marks as per the reviewers’ comments. Our point-by-point responses to the reviewers’ comments are given below.

We believe that the revision has substantially improved our manuscript and hope that the revised version is now suitable for further review.

Thank you for considering our work. We eagerly await your response.

Sincerely,

Fuu-Jen Tsai

China Medical University

No. 2, Yuh-Der Road, Taichung, Taiwan 40442

+886 4 22062121

[000704@tool.caaumed.org.tw](mailto:000704@tool.caaumed.org.tw)

**Responses to comments by Reviewer 1:**

Lin et al. have conducted a GWAS analysis on height and FSS in a Han Taiwanese cohort. They further employed 5 East Asian Biobanks to access genetic correlations. They also performed a PheWAS and PRS analysis. They report genetic associations height and FSS and pleiotropic effects with AF and endometriosis. The results are not novel, as the reported loci are already reported before in larger GWAS studies and so are the PheWAS results for AF and endometriosis. The novelty of this manuscript is the genetic diversity, as the study is focusing on East Asian populations. However, the full potential of this study has not been realised here, as there is no attempt for a meta-analysis across the non-overlapping East-Asian Biobanks for available phenotypes. The results mainly focus on the CMUH cohort and only the genetic correlations consider the other biobanks individually. This results in underpowered conclusions. Furthermore, the methods are poorly described making the results difficult to interpret. In addition, tables and figures are not always informative and the result section is not expanded in enough detail. Discussion is also brief and some of the conclusions are not corroborated by the results.

We thank the reviewer for the constructive feedback. The manuscript has been revised and clarified point by point as outlined below, with all changes highlighted in the updated version.

We conducted a meta-analysis across Han Taiwanese and Korean cohorts, with associations further replicated in CMUH and BBJ. Height meta-analysis PRSs were significantly associated with atrial fibrillation in both CMUH and BBJ cohorts and with endometriosis in CMUH only (**S27 Table**), and survival analyses confirmed significantly higher AF risk and cumulative incidence in the highest PRS quintiles (**S14 Fig**). These findings highlight the value of meta-analytic approaches where feasible and underscore the broader clinical relevance of stature genetics.

We have expanded and clarified the description of phenotyping, cohort characteristics, ICD coding, PRS analyses, Cox models, and locus definition procedures, as well as the MAGMA workflow (**Materials and methods, pages 15–20, lines 368–482**), with new supplementary tables added (**S1–S39 Tables**).

We revised several figures and legends, including replacing dots with up/down triangles in **Figure 4** and using the middle PRS group as the reference in **Figure 5** and **S14 Fig**, along with an expanded description of the survival curves (**Results, page 12, lines 270–283**).

The Discussion has been expanded and revised to better align with the results (**Discussion, pages 12–15, lines 286–366**). Specifically, we distinguished known from novel loci, incorporated pathway analyses, and corrected overstated claims regarding onset age and endometriosis. We also added key epidemiologic references to provide fuller context, ensuring that the conclusions are now fully supported by the findings.

**Major points:**

1. Please provide information on how phenotypes were measured/defined in the CMUH cohort, what was the age range and the length of follow-up. A supplementary table with the descriptives would be informative. Please also provide the ICD10 codes used to define FSS.

We thank the reviewer for this helpful comment. In the CMUH cohort, disease phenotypes were defined using PheCODEs mapped from ICD-9-CM diagnoses [1], with follow-up from birth until the first PheCODE-defined disease or the last data entry (**S23 and S25 Tables**). Cases were defined as ≥2 outpatient visits or one inpatient admission, and diseases with fewer than 200 cases were excluded.

The cohort had a mean age of 47 years and an average follow-up of 46 years, with descriptive characteristics provided in **S23 and S25 Tables**. Familial short stature (FSS) was defined using ICD9-CM code 783.43 and ICD10-CM code R62.52 (**S25 Table)**. We have revised the manuscript accordingly (**Results, page 9, lines 209–215; page 10, lines 222–229**).

2. Please describe how lifestyle information was collected and quantified and the rationale behind residualizing the height with lifestyle adjustments before performing the GWAS. Please also explain whether the age adjustment refers to the age of recruitment or the age of the height measurement was used.

We thank the reviewer for this insightful comment. In the Taiwan Biobank (TWB), lifestyle information (drinking, smoking, exercise) was collected via standardized questionnaires at recruitment, along with physical measurements including height and blood sampling (https://www.twbiobank.org.tw/new_web/).

Thus, the age used in our analysis refers to the age at recruitment, when height was measured. Our GWAS model was adjusted for age, sex, and the top 10 genetic principal components, but not for lifestyle variables. We have revised the manuscript to clarify this (**Materials and Methods, page 16, lines 380–384**).

3. Please describe the PRS analysis and cox models in more detail.

We thank the reviewer for the insightful comment. Accordingly, we expanded the **Materials and Methods** to include additional details on PRS construction and Cox proportional hazards modeling (**page 19, lines 449–459; page 20, lines 474–481**).

4. Please describe how height and FSS loci were defined from the GWAS data and how the MAGMA analysis was performed.

We appreciate the reviewer’s constructive comments. Height- and FSS-associated loci were defined as distinct regions represented by lead SNPs reaching genome-wide significance (*P* < 5 × 10⁻⁸) and identified using LocusZoom (<http://locuszoom.org/>) **(S2 and S12 tables)**.

We then performed gene-based GWAS in MAGMA (v1.06) within SNP2GENE, mapping SNPs to protein-coding genes using Ensembl identifiers with default windows and applying the SNP-wise mean model. Significant genes were identified at a Bonferroni threshold of *P* < 0.05/18,651, with results summarized in gene-based GWAS tables **(S5 and S15 tables).** Corresponding revisions have been added to the manuscript (**Results, page 7, lines 153–155; page 8, lines 186–187**) (**Materials and Methods, page 18, lines 431–438**).

5. It is unclear why a meta-analysis for available, non-overlapping biobanks was not attempted. The authors need to comment on that. Line 134 reports a total of 1.2M East Asians included in this study, which is quite misleading as the cohorts were analyzed individually across this study.

We thank the reviewer for this comment. We conducted a meta-analysis of Han Taiwanese and Korean cohorts, identifying 433 loci and 1,690 genes, including 1,563 from GWAS annotations and 967 from gene-based analyses (**S35-S37 Tables**) (**S10 and S11 Figs**). Key associations were observed between meta-analysis–derived height PRSs and atrial fibrillation across both CMUH and BBJ cohorts, whereas the association with endometriosis was replicated in CMUH only (**S27 Table**), and survival analyses showed that individuals in the highest PRS quintile had significantly increased AF risk and cumulative incidence (**S14 Fig**). Corresponding revisions have been added to the manuscript (**Results, page 10, lines 230–236; page 12, lines 282–283**) (**Discussion, page 13, lines 294–296; page 14, lines 333–339**).

The misleading statement of ‘1.2M East Asians’ has been removed and revised to ‘*encompassing individuals of Han Taiwanese, Han Chinese, Japanese, and Korean ancestries*’ (**Results, page 6, line 134**).

6. Please discuss how the current GWAS in the CMUH cohort differs from your previous published GWAS for height in the same cohort: https://doi.org/10.1186/s12916-022-02450-w

We thank the reviewer for raising this point. Our previous GWAS for height in CMUH was based on the CMUH_250K_SNP dataset (2018–2021) and used PRSice-2 for PRS construction (<https://choishingwan.github.io/PRSice/>), whereas the current study utilized the expanded CMUH_410K_SNP dataset (2018–2023) (**S3 Fig**) and applied PRS-CS with the 1000 Genomes Phase 3 East Asian LD reference panel [2]**.**

In addition, the previous GWAS analysis included 67,452 TWB participants, while the present GWAS analyzed 120,301 individuals (**S1 Fig**).

The earlier study highlighted associations of genetically determined height with anthropometric and metabolic traits. Here, we extend these findings by replicating associations in the CMUH and BBJ cohorts (**S27 Table**), where height-derived polygenic risk scores (PRSs) were consistently associated with atrial fibrillation across both biobanks and with endometriosis in CMUH only. Survival analyses revealed that individuals in the highest body height and meta-analysis PRS quintiles had significantly elevated AF risk and cumulative incidence (**Figure 5 and** **S14 Fig**). Corresponding revisions have been added to the manuscript (**Discussion, page 14, lines 326–333**).

7. Tables S1 and S3 are missing the effect and other allele, freq, n, beta and se stats. Please add.

We thank the reviewer for this helpful suggestion. We have revised **S2, S12, and S35 Tables** to include the reference and alternative alleles, allele frequency, sample size (n), beta, standard error (SE), and *P*-value statistics.

8. Please explain the analysis used to derive the results included in Tables S2 and S4. Please also explain what the NAs are in that Tables; many of them reported for the “novel” genes. Please explain how novel genes were defined. Are these supposed to be causal genes? The methods for this analysis are missing.

We thank the reviewer for this constructive comment. Previously, **Tables S2 and S4** combined SNP-based gene annotations and MAGMA gene-based results in FUMA. For clarity, these analyses are now separated: SNP-based annotations in **S4, S14, and S36 Tables**, and MAGMA gene-level results in **S5, S15, and S37 Tables**.

Novelty was defined only at the locus level by: (1) genome-wide significance in our GWAS but not in comparison ancestries, (2) colocalization evidence for population-specific signals, and (3) absence of the nearest gene from SNP- and gene-based lists, yielding four novel loci for height and none for FSS. As MAGMA aggregates SNP associations to genes, a few variants can implicate multiple genes through positional, eQTL, or chromatin mapping. Because such signals reflect regulatory complexity rather than true novelty, we define novelty only at the locus level, not at the gene level. These revisions are highlighted in the **Results (page 7, lines 158–164; page 8, lines 189–191)** and **Materials and methods (page 18, lines 419–438**).

9. Table S5 and S6: significance should be defined at a multiple testing not nominal significance. Several non-standard abbreviations are used in these tables but not explained.

We thank the reviewer for these helpful comments. In the revised version, statistical significance in **S21 and S22 Tables** is now defined using Bonferroni correction, and all non-standard abbreviations have been clarified.

10. Table S8: endometriosis should not be significant based on the Bonferroni threshold?

We thank the reviewer for pointing this out. Endometriosis was a nominal association (*P* = 2.72 × 10⁻⁴) that did not meet the Bonferroni threshold. We have clarified this in **S26 Table** and in the text, indicating it as nominal but not genome-wide significant (**Results, page 10, lines 227–229**).

11. Figure 1 is misleading as it is not clear that the other 5 biobanks are ONLY used for the genetic correlation part.

We thank the reviewer for this observation. We have clarified that GWAS of height and FSS were conducted only in TWB and CMUH, while the other five East Asian biobanks were used solely for cross-trait genetic correlation analyses. **Figure 1**, its legend, and the text (**Results, page 6, lines 134–135**) have been revised accordingly.

12. Figure B and E: it is unclear how these plots were generated.

We thank the reviewer for this observation. We clarified that **S2 and S5 Figs** were generated from gene-based GWAS using MAGMA within FUMA (<https://fuma.ctglab.nl/snp2gene>(https://fuma.ctglab.nl/snp2gene), which aggregates SNP-level associations into gene-level signals. Significant genes were defined at a Bonferroni threshold (*P* < 0.05/18,651), with details provided in the revised **Materials and methods** (**page 18, lines 431–438**).

13. Figure 2C,F and Figure 3 seems to present the same results.

We thank the reviewer for the opportunity to clarify. The updated **Figures 2B and 2D** listed traits genetically correlated with height and FSS, respectively, whereas **Figure 3** applied hierarchical genetic clustering to these results, revealing biologically meaningful trait groupings. These revisions are highlighted in the **Results (page 7, line 167; page 8, line 194; page 9, lines 200–201**).

14. Figure 4: please make sure all text is readable, and the dots are replaced with up/down triangles to highlight the direction of effect.

We thank the reviewer for the suggestion. **Figure 4** and its legend were revised for clarity, replacing dots with up/down triangles to indicate effect direction.

15. Figure 5: A fairer comparison would be to set the middle PRS group as the ref. The right-hand side plots are not described in the results. What are the different age points where the curves diverge and are these significantly different?

We thank the reviewer for the suggestion. **Figure 5 and S14 Fig** have been revised to use the middle PRS group as the reference, updating the figures and legends accordingly.

The Results section has now been revised to explicitly describe the right-hand side plots of **Figure 5**. Specifically, we added the following text: *“The right panel showed that cumulative incidence curves diverged once incidence reached 10%, with high-risk individuals (PRS_80–100%_) reaching this at age 78.2 versus 81.6 in low-risk individuals (PRS_0–20%_). The divergence was statistically significant across groups, confirmed by the log-rank test (High vs Low: P = 5.48E-11; High vs Middle: P = 3.05E-02; Middle vs Low: P = 1.11E-05), with high-PRS individuals experiencing earlier and greater incidence. These findings were consistent with survival analyses of meta-analysis–derived PRSs (****S14 Fig****).”* (**Results, page 12, lines 278-283**).

As shown in **Figure 5 (right panel)**, at the 10% incidence threshold cumulative incidence curves diverged, with high-risk individuals (PRS_80–100%_) reaching this at age 78.2 versus age 81.6 in low-risk individuals (PRS_0–20%_). The differences across groups were statistically significant, as confirmed by the log-rank test (High vs Low: *P* = 5.48E-11; High vs Middle: *P* = 3.05E-02; Middle vs Low: *P* = 1.11E-05). Consistently, survival analyses of meta-analysis–derived PRSs confirmed increased AF risk and cumulative incidence in the highest quintile (**S14 Fig**).

16. In the Discussion, the authors claim that: “Herein, we identified 293 loci for body height and 5 for FSS in Han Taiwanese, revealing both overlapping and distinct genetic pathways.” The authors need to clearly state how many known and novel loci. In addition, there is no evidence of pathway analysis here, so the last part of this statement is not supported.

We thank the reviewer for the comment. In our GWAS of 120,301 Han Taiwanese individuals, we identified 293 loci for height, of which four (*NSD2, AL049612.1, AC133634.1*, and *RBM4*) were defined as novel using a stringent three-step framework (**Results, page 7, lines 158-164**). For FSS, we identified five loci, but none met criteria for novelty (**Results, page 8, lines 189-191**).

Pathway analyses highlighted biological processes including cell cycle G1/S checkpoint regulation and growth hormone signaling, supporting the interpretation of the overlapping genetic pathways (**Discussion, page 13, lines 305-307**). Accordingly, the Discussion sentence was revised to: *“We identified 293 loci for body height and 5 for FSS in Han Taiwanese, including four novel height loci, highlighting shared genetic pathways such as cell cycle G1/S checkpoint regulation and growth hormone signaling”* (**Discussion, page 12, lines 287-289**).

17. In the Discussion the authors claim that: “Our findings reveal unique genetic factors influencing stature in Han Taiwanese” but all of the GWAS hits were previously reported. How are there unique genetic factors?

We thank the reviewer for this point. In our GWAS of 120,301 Han Taiwanese individuals, we identified 293 loci for height, including four novel loci (*NSD2, AL049612.1, AC133634.1, RBM4*) defined by a stringent three-step framework (**Results, page 7, lines 158–164**). For FSS, five loci were identified, but none were novel (**Results, page 8, lines 189–191**). The **Discussion** has been revised accordingly (**pages 13, lines 307–312**).

18. In the Discussion the authors claim that: “Herein, GWAS, PheWAS, and PRS survival analyses were performed, revealing that genetically taller stature is linked to increased risks of AF and endometriosis, whereas shorter stature is associated with later onset and a reduced risk of endometriosis individuals”. This statement is not fully corroborated by the results, since the age of onset was not properly tested in the analysis. Furthermore, endometriosis was actually not Bonferroni significant with FSS.

We thank the reviewer for this comment. We revised the **Discussion** to remove the “later onset” claim and clarified that endometriosis did not reach Bonferroni significance (**Discussion, page 15, lines 361–363**). The **Discussion** now states: *“Herein, GWAS, PheWAS, and PRS survival analyses were performed, revealing that genetically taller stature is linked to an increased risk of AF, whereas no significant association was observed with endometriosis after correction for multiple testing.”*.

19. Several key references reporting the association between low height and endometriosis are not included or discussed in this paper.

We thank the reviewer for this comment. We have added a paragraph in the **Discussion** to incorporate key epidemiologic studies on stature and endometriosis. These report that taller stature and earlier menarche increase the risk of endometriosis [3, 4], whereas other studies suggest childhood obesity may reduce risk [5], despite its link with earlier menarche [6]. Our MR analyses found no direct causal effect of height or FSS, but associations emerged after adjusting for menarche or body weight, indicating that these traits exert stronger influences on risk than stature itself. These revisions clarify the complexity of the relationship and ensure that key literature is now discussed (**Discussion, page 15, lines 345–353**).

**Responses to comments by Reviewer 2:**

In “Unraveling the genetic links between stature and disease in East Asians: A multibiobank genetic correlation and risk prediction study”; the authors perform a wide-ranging analysis of the health risks associated with height, and additionally explore the risks associated with familial short stature, in previously under-studied east-asian populations. They perform GWAS, PheWAS and genetic correlation analyses and MAGMA gene analyses.

We thank the reviewer for the constructive feedback. **(1)** To strengthen generalizability, we replicated key associations in CMUH and BBJ, confirming consistent effects of height and FSS (**Results, page 10, lines 230–236**) (**Discussion, page 14, lines 333–336**). **(2)** We distinguished loci and gene-level findings using SNP-based and MAGMA analyses. Novel loci were defined by genome-wide significance, colocalization evidence (PP.H3.abf and PP.H4.abf ≤ 0.7) across BBJ, KoGES, and Yengo et al. (2022) [7-9], and absence of the nearest gene from SNP- and gene-based lists, yielding four novel loci for height and none for FSS (**Results, page 7, lines 158–164; page 8, lines 189–191**) (**Materials and methods, page 18, lines 419–438**). **(3)** We reported biobank-wide genetic correlations using stringent thresholds to control for multiple testing (**S6-S10 Tables**) (**S16-S20 Tables**). **(4)** Mendelian randomization showed that height–endometriosis associations are mediated by menarche and body weight rather than direct effects (**Results, page 11, lines 245–258**) (**Discussion, page 15, lines 345–353**). **(5)** Minor revisions included updating terminology (“puberty”), adding references, clarifying height/FSS-specific groupings, presenting cross-biobank trait correlations, and rephrasing “lower risk” as “protective effects. Together, these revisions enhance clarity and rigor, underscoring how our East Asian multi-biobank study complements predominantly European findings.

1. Overall, whilst I think the study concept is good, I think there are improvements to be made. Whilst I found the links to different health risks interesting, I would either like to see some analysis of other ancestries, to see if the association is unique or altered within the populations studied here, or a greater discussion on the literature for this issue.

We thank the reviewer for this valuable comment. To evaluate generalizability, we replicated key associations using stature- and GWAS meta-analysis–derived PRSs in both the CMUH and BBJ cohorts. Consistent associations were observed between height-derived PRSs and atrial fibrillation (CMUH: OR = 1.12, 95% CI = 1.09–1.15; BBJ: OR = 1.14–1.15, 95% CI = 1.12–1.17), and between height PRSs and endometriosis in CMUH (OR = 1.07, 95% CI = 1.05–1.10) (**S27 Table**). In contrast, familial short stature (FSS)–derived PRSs showed no significant association with endometriosis in BBJ. These cross-population analyses indicate that the height–AF associations are robust and not restricted to a single ancestry. These revisions are highlighted in the **Results (page 10, lines 230–236)** and **Discussion (page 14, lines 333–336)**.

2. I found it odd that none of the genetic loci were classified as new, but the MAGMA analysis highlighted new genes? This is particularly noticeable in ST3: you identify three single variants on chromosome 12, which you link to 20 separate genes in ST4. Can you justify this more clearly?

We apologize for the confusion between novel loci and newly implicated genes. Previously, **Tables S2 and S4** combined SNP-based gene annotations and MAGMA gene-based results in FUMA. For clarity, these analyses are now separated: SNP-based annotations in **S4, S14, and S36 Tables**, and MAGMA gene-level results in **S5, S15, and S37 Tables**.

Novelty was defined only at the locus level by: (1) genome-wide significance in our GWAS but not in comparison ancestries, (2) colocalization evidence for population-specific signals, and (3) absence of the nearest gene from SNP- and gene-based lists, yielding four novel loci for height and none for FSS. As MAGMA aggregates SNP associations to genes, a few variants can implicate multiple genes through positional, eQTL, or chromatin mapping. Because such signals reflect regulatory complexity rather than true novelty, we define novelty only at the locus level, not the gene level. These revisions are highlighted in the **Results (page 7, lines 148–164; page 8, lines 182–191)** and **Materials and methods (page 18, lines 419–438**).

3. As an aside, it would be beneficial to check your criteria for defining a novel loci. If I understand correctly, you consider a loci as previously reported if there is a GWAS catalog hit within 1MB? I think this is quite conservative – could you do some co-localisation analysis? It may be that your loci is distinct to previous loci despite being proximal? Or your SNP is a better representation of the loci within EAS? Broadly, throughout the article, I also found the lack of discussion and comparison with the Yengo et al 2022 meta-analysis of 5M individuals for height quite jarring.

We thank the reviewer for this insightful comment. As suggested by the reviewer, we performed colocalization analysis using the coloc R package (<https://cran.r-project.org/web/packages/coloc/vignettes/a01_intro.html>) [10].

Novel loci were defined using a three-step framework using a three-step framework combining cross-ancestry significance testing, colocalization analyses with BBJ, KoGES, and Yengo et al. (2022) EUR/EAS data [7-9] (retaining loci only when PP.H3.abf and PP.H4.abf ≤ 0.7) (**S3 and S13 Tables**), and exclusion of the nearest gene from prior SNP annotated- or gene-based GWAS-identified gene lists (**S4 and S5 Tables**), yielding four novel loci for height and none for FSS. These revisions are reflected in the **Results** (**page 7, lines 158–164**; **page 8, lines 189–191**) and **Discussion** (**page 13, lines 307–309**).

4. For the genetic correlations, I wonder whether a more targeted approach would be good, to lower your multiple-testing threshold?

We appreciate the reviewer’s suggestion of a targeted approach to reduce the multiple-testing burden. However, our aim was a comprehensive discovery-oriented screen across five East Asian biobanks, we did not restrict analyses to targeted traits but instead applied stringent significance thresholds to control for multiple testing (**S6-S10 Tables**) (**S16-S20 Tables**).

5. I note that you found genetic correlation between height (and FSS) and endometriosis. However, as you state in the introduction, height is associated with menarche, which is further associated with reproductive health outcomes. I think more needs to be done to demonstrate the causal nature of this relationship, such as by adjusting for the genetic architecture of menarche.

We thank the reviewer for highlighting the potential mediating role of age at menarche in the relationship between height and endometriosis. Epidemiologic studies have reported complex and sometimes inconsistent associations among stature, menarche, and endometriosis [3-6]. As suggested, we conducted Mendelian randomization (MR) analyses, which showed no direct causal effect of height on endometriosis (*P* > 0.05/5; **S8A Fig, left; S29–S31 Tables**). Instead, associations became significant only after adjusting for age at menarche (MVMR-Lasso: OR = 1.112, *P* = 9.07 × 10⁻³) or body weight (OR = 1.165, *P* = 4.63 × 10⁻³; **S8B–S8C Fig, left**), indicating mediation by these traits.

Similarly, FSS effects emerged only after body weight adjustment (MVMR-Robust: OR = 0.903, *P* = 6.68 × 10⁻³; **S8A–S8C Fig, right**). These results suggest that the observed correlations are largely explained by growth, reproductive, and metabolic factors rather than direct effects of height or FSS. We have revised the **Results** (**page 11, lines 245–258**) and **Discussion** (**page 15, lines 345–353**) accordingly.

**Minor comments:**

1. Line 101 – I don’t think its accurate to say height growth ‘concludes at menarche’, as males don’t undergo menarche.

We thank the reviewer for this clarification. We have revised the text to state ‘puberty’ instead of ‘menarche’ to more accurately describe the conclusion of height growth (**Introduction**, **page 5, line 100**).

2. Line 101 – Yengo et al 2022 should to be discussed here.

We thank the reviewer for this suggestion and have included the Yengo et al. (2022) reference in the revised text (**Introduction**, **page 5, line 102**).

3. Line 109 – reference for ‘most common form of short stature’.

We have added the appropriate references for the statement on ‘the most common form of short stature (**Introduction**, **page 5, line 109**).

4. Line 139 – I note you classify BMI as an FSS-specific trait. Recent articles (e.g. PMID 39818621) have discussed how ratio traits such as BMI are affected by both components of that ratio. It would be nice to see more justification overall of how you split traits into height/FSS specific groupings.

We thank the reviewer for this valuable comment. While BMI is a ratio trait influenced by both weight and height, our genetic correlation analyses showed it was significantly correlated with FSS but not with height after multiple-testing correction (**S6-S10 Tables**) (**S16-S20 Tables**) (**Figs 2B and 2D**). We have clarified this rationale in the revised text (**Results**, **page 7, line 167; page 8, line 194**).

5. Line 152 & Figure 2 – Ideally I’d like to see the correlations across all the biobanks analysed for any trait which shows a significant correlation in one biobank.

Thank you for the helpful suggestion. We now present the correlations across all biobanks for traits showing significant associations in any biobank, as shown in the updated **Figure 3**, which compares Rg values and significance across biobanks.

6. Line 211 & 213 ‘lower risk of endometriosis’ – I would suggest rephrasing these statements throughout the manuscript to ‘protective effects’, to avoid confusion.

We have revised the wording to “protective effects” throughout the manuscript as suggested.

**Responses to comments by Reviewer 3:**

This manuscript by Lin et al. provides a valuable contribution to our understanding of the genetic architecture of height, particularly in East Asian populations. The effort to integrate multiple large-scale biobanks across East Asia to explore the genetic underpinnings of stature and its potential links to disease is commendable. However, I don’t think the manuscript itself is well polished (as well as the figures), which makes it difficult to follow. The transparency of the analyses is another great concern. The manuscript requires substantial revision to improve its readability and methodological transparency. In addition to this, I have the following specific comments:

We thank the reviewer for the constructive feedback. We clarified trait categorization (**Figure 1**), revised figure titles and legends, and provided full phenotype lists with Bonferroni correction details (**S6-S10 Tables**) (**S16-S20 Tables**). We added demographic details for TWB5 and CMUH GWAS participants (**S1 and S4 Figs**) (**S1 and S11 Tables**), and expanded the Methods to include genotyping, imputation, QC, phenotypic variance explained, genomic inflation, and additional analyses (FUMA, MAGMA, PRS-CS, coloc, survival analyses) (**Materials and methods, pages 15-20, lines 368-481**). Finally, we acknowledged the need for functional follow-up of novel loci and highlighted this limitation in the **Discussion** (**Page 15, lines 356-357**). These revisions improve clarity, transparency, and rigor of the manuscript.

1. The authors conducted cross-trait genetic correlation analyses across five East Asian biobanks, selecting and categorizing a wide range of traits. I’m curious about the rationale and justification for this categorization (i.e., height specific, shared, and FSS specific). Additionally, I was unable to find a table listing all the phenotypes included in the genetic correlation analyses.

We appreciate the reviewer’s insightful comment. The categorization of traits into height-specific, shared, and FSS-specific was guided by their biological and clinical relevance to height and FSS, as illustrated in **Figure 1**. Because our aim was to conduct a comprehensive, discovery-oriented screen across five East Asian biobanks, we classified significant genetic correlations (after multiple testing correction) as height-specific (observed only with height), shared (observed with both height and FSS), or FSS-specific (observed only with FSS). We have also added supplementary tables listing all phenotypes included in the cross-trait genetic correlation analyses (**S6-S10 Tables**) (**S16-S20 Tables**).

2. Fig 2A-B: The title of each plot is not clear to me, what do the numbers after the cohort names (e.g., TWB5, FSScontr27) represent?

We thank the reviewer for this comment. The numbers following the cohort names indicate the dataset version (e.g., TWB5 denotes Taiwan Biobank, version 5). We have revised the manuscript and figures to consistently use “FSS” in the title and text for clarity.

3. Fig 2C,F: Here the P-value column refers to the value after multiple testing correction? And what method was used for this (FDR, Bonferroni?), I could not find it in the Methods section.

We thank the reviewer for raising this point. The *P*-value columns in the updated **Figure 2B and 2D** refer to values after multiple testing correction using the Bonferroni method. This information has now been clarified (**Results**, **page 7, line 167; page 8, line 194**). All phenotypes analyzed are now listed in the supplementary tables (**S6-S10 Tables**) (**S16-S20 Tables**).

4. There is a lack of reporting the demographics for the population selected for the GWAS analysis.

We thank the reviewer for pointing out the need to provide clearer demographic details. For the height GWAS in TWB5, after applying quality control filters, we analyzed 120,301 participants (mean age 49.6 ± 11.1 years; 62.5% female) with an average body height of 162.2 ± 8.3 cm (**S1 Fig**) (**S1 Table**).

For the FSS GWAS, we analyzed data from the CMUH Biobank, which included 2,050 cases (mean age 11.1 ± 5.4 years; 50.0% male) and 27,966 controls (mean age 44.4 ± 15.1 years; 45.4% male), showing significant age and sex differences (*P* < 0.001) (**S4 Fig**) (**S11 Table**). We have added these details to the revised manuscript to ensure clearer reporting of the study populations (**Results**, **page 6, lines 143-145; page 8, lines 173-178**).

5. The authors failed to provide some major details for each step of analysis in the Methods section. For instance, there is a lack of details on genotyping, imputation and quality control. For instance, what is the phenotypic variance explained by the identified loci? Have the authors calculated the genomic inflation?

We thank the reviewer for this important suggestion and have expanded the Methods section to include full details on genotyping, imputation, and both SNP- and individual-level quality control using the Axiom TPM v1 array and BEAGLE-based imputation with TWB WGS as the reference [11, 12] (**Materials and methods, page 16, lines 375-382; pages 16-17, lines 387-398**).

We now report the phenotypic variance explained by the identified loci (**S2 and S12 Tables**) and genomic inflation metrics for all phenotypes **(S6–S10, S16–S22 Tables**), noting that LDSC intercepts indicate minimal confounding despite modest λGC values.

We also added details on SNP-based annotation (FUMA), gene-based analyses (MAGMA), PRS construction (PRS-CS), colocalization (coloc), and survival analyses (Cox regression and Kaplan–Meier curves) (**Materials and methods, pages 18-20, lines 419-481)**. These revisions improve clarity, transparency, and reproducibility.

6. There is a lack of functional follow-up in novel loci identified for the East Asian population.

We thank the reviewer for this important point. While we identified novel loci in the East Asian population, we acknowledge that functional studies are required to validate their roles in growth biology and long-term health outcomes, and we have highlighted this limitation in the revised **Discussion (Page 15, lines 356-357)**.

**Responses to comments by Reviewer 4:**

The authors conducted new GWAS in the Han Taiwanese population for height and familial short stature, and explored genetic correlations with phenome-wide outcomes. They confirmed the previously established associations between tall stature and increased risk for atrial fibrillation (AF) and endometriosis. Furthermore, they identified that the height PRS was significantly associated with AF and endometriosis risk in the East Asian population. The analysis plan is solid, and the findings are robust. My comments are below.

We thank the reviewer for the helpful comments. We appreciate these encouraging remarks and address the reviewer’s specific comments: **(1)** Mendelian randomization showed no direct effect of height on endometriosis, with mediation by menarche and body weight (**Results, page 11, lines 245–258**) (**Discussion, page 15, lines 345–353**). **(2)** Height PRS provided additional predictive value for AF but not for endometriosis (**Results, page 10, lines 237–243**). **(3)** FSS was strongly negatively correlated with height (rg ≈ −0.80 to −0.95; **Fig. 2D**; **S16–S20 Tables**), with effect sizes for the five FSS loci reported in **S12 Table**. **(4)** We revised the abstract to specify associations with height PRS and FSS PRS, and described FSS PRS findings as showing “protective effects” for consistency (**Abstract, page 3, line 63**) and revised PRS effect sizes for interpretability (**Fig. 5**), and corrected novelty definitions, phenotyping, and data availability. Together, these revisions improve clarity and highlight the contribution of our East Asian multi-biobank study.

**Major comments**

1. In clinical epidemiology, endometriosis is associated with early menarche and taller stature. However, it is well established that taller stature is generally associated with later menarche. This genetic analysis appears to recapitulate that pattern as well. This seems discrepant, as one would expect the pathway to be: early menarche, shorter stature, higher risk of endometriosis. Are there any explanations for this discrepancy? Could genetics contribute to this paradox?

We thank the reviewer for highlighting this important paradox. Epidemiologic studies have reported complex and sometimes inconsistent associations between stature, menarche, and endometriosis [3-6]. As suggested, we conducted Mendelian randomization (MR) analyses, which showed no direct causal effect of height on endometriosis (*P* > 0.05/5; **S8A Fig, left; S29–S31 Tables**). Instead, associations became significant only after adjusting for age at menarche (MVMR-Lasso: OR = 1.112, *P* = 9.07 × 10⁻³) or body weight (OR = 1.165, *P* = 4.63 × 10⁻³; **S8B–S8C Fig, left**), indicating mediation by these traits.

Similarly, FSS effects emerged only after body weight adjustment (MVMR-Robust: OR = 0.903, *P* = 6.68 × 10⁻³; **S8A–S8C Fig, right**). These results suggest that the observed correlations are largely explained by growth, reproductive, and metabolic factors rather than direct effects of height or FSS. We have revised the **Results** (**page 11, lines 245–258**) and **Discussion** (**page 15, lines 345–353**) accordingly.

2. The authors claim that the height PRS has utility in predicting AF and endometriosis. I was curious whether the height PRS captures genetic variance not explained by the AF-PRS or endometriosis PRS, which would support its additional value in predicting these two outcomes.

We thank the reviewer for this insightful suggestion. To assess whether the height PRS adds predictive value beyond disease-specific PRSs, we performed conditional multivariate logistic regression including both the height PRS and the respective AF or endometriosis PRS (**S28 Table**).

The height PRS remained significantly associated with atrial flutter/fibrillation even after adjusting for the AF PRS (**Model 2**: OR = 1.09, 95% CI: 1.06–1.12, *P* = 1.43×10⁻¹¹), supporting additional predictive value. In contrast, its association with endometriosis was no longer significant after adjustment for the endometriosis PRS (**Model 2**: OR = 1.01, 95% CI: 0.98–1.04, *P* = 0.45), suggesting no independent contribution. We have revised the **Results** accordingly **(page 10, lines 237–243**).

3. The authors reported genetic correlations between height and the phenome, as well as between FSS and the phenome. I was particularly interested in the degree of negative genetic correlation between height and FSS. Additionally, I was curious about the height effect sizes of the five loci associated with FSS.

We thank the reviewer for the valuable comment. Across five East Asian biobanks, FSS showed a consistently strong negative genetic correlation with body height (rg ≈ −0.80 to −0.95, all significant) (**S16–S20 Tables**) (**Figure 2D**), confirming a robust inverse genetic relationship.

The five loci associated with FSS (*DIS3L2, NCAPG, ATF7, CS,* and *IGF1*) overlap with known body height GWAS loci. The corresponding height effect sizes are provided in **S12 Table**.

4. In the abstract, the sentence "PheWAS further showed that tall stature increased risks of AF and endometriosis, while short stature reduced endometriosis risk." After reading through the manuscript, I understood that the authors tried to present that high height PRS is associated with increased risk for AF and endometriosis, but the PRS for FSS was associated with lower risk for endometriosis. However, this is a bit difficult to understand at the first glance. As associations between high-height PRS and high endometriosis risk and high-FFS PRS and low endometriosis risk are indicating the same direction of associations, please reconsider the expression here.

We thank the reviewer for this helpful suggestion. We have revised the abstract to clarify that associations refer specifically to height PRS and FSS PRS, and now describe the FSS PRS findings as showing ‘protective effects’ for consistency (**Abstract, page 3, line 63**).

**Minor comments**

5. Line 104: The authors refer to "the risk of short stature," but this phrasing may not be entirely neutral. Since short stature itself is not necessarily a pathological condition, and to avoid potential stigma, we recommend using more neutral language.

We thank the reviewer for noting that “at risk of short stature” may imply pathology. We have revised the phrasing to *“Polygenic risk scores (PRS) for adult height have been demonstrated to be as predictive as mid‐parental height for identifying children with lower expected height”* to ensure neutral, non-stigmatizing language (**Introduction, page 5, lines 103–104**).

6. line 189 "A higher genetically determined short stature was linked to an increased risk of clinical short stature and reduced risks of endometriosis and atopic/contact dermatitis" is hard to interpret. Please reconsider rephrasing.

Thank you for the valuable comment. Accordingly, we have revised the sentence to *“In the CMUH cohort, PheWAS of the short-stature–specific PRS identified 81 disease phenotypes (P < 0.05), with strongest associations in abnormal growth and short stature, and nominal associations in endometriosis (****Fig 4B****) (****S26 Table****)”* for improved clarity (**Results, page 10, lines 227–229**).

7. Line 197 "Individuals in the highest PRS quintile showed significantly higher risks of AF (adjusted hazards ratio[HR]: 1.31, P = 6.76x10-12) and endometriosis (adjusted HR: 1.13, P = 1.45x10-3) and correspondingly higher cumulative incidence rates (AF: P < 0.0001; endometriosis:P = 0.0011) (Fig 5A)." The authors report hazard ratios comparing the top and bottom quintiles of the PRS, which inflates the HR and limits interpretability. For example, this is akin to comparing disease risk between individuals under 20 and over 80 years old. A more interpretable estimate would compare the top 20% to the rest of the population. Also, reporting effect sizes by 1SD-PRS is helping interpretation and improve comparability of the results.

We thank the reviewer for this insightful suggestion. Accordingly, we have revised **Figure 5 and S14 Fig** to use the middle PRS quintile as the reference category. Using height PRS as the exposure, AF risk was elevated in the highest quintile versus the middle quintile (HR = 1.09, *P* = 1.72 × 10⁻²), in the top 20% versus the remaining population (HR = 1.16, 95% CI: 1.10–1.23, *P* = 2.70 × 10⁻⁷), and per SD-PRS (HR = 1.10, 95% CI: 1.07–1.13, *P* = 9.80 × 10⁻¹⁵) (**Figure 5, left panel**). Similar results using height meta-analysis PRS as the exposure, AF risk was elevated in the highest quintile versus the middle quintile (HR = 1.17, *P* = 2.40 × 10⁻^5^), in the top 20% versus the remaining population (HR = 1.21, 95% CI: 1.14–1.28, *P* = 7.34 × 10⁻^11^), and per SD-PRS (HR = 1.11, 95% CI: 1.09–1.14, *P* = 1.16 × 10⁻¹^8^) (**S14 Fig, left panel**). The Results and Discussion have been updated accordingly (**Results, page 12, lines 272–277) (Discussion, page 14, lines 336–339**).

8. Line 214 "Overall, these findings highlighted the strong genetic associations between stature and major health outcomes in East Asians..." This might be overstating the utility of the study. While AF and endometriosis are certainly important outcomes, they do not represent major health outcomes as a whole.

We thank the reviewer for this important point. To avoid overstating the implications, we have revised the sentence to: *“These findings suggest genetic links between stature and selected health outcomes in East Asians”* **(Discussion, page 13, lines 296–297**).

9. Line 219 "Gene-based analysis using MAGMA identified 15 novel genes associated with body height (S2 Table)." I could not find the definition of "Novel" throughout the manuscript. Were not these genes identified in the large scale GWAS (ex. PMID: 36224396)? If so, how this novelty was determined.

We apologize for the confusion between novel loci and newly implicated genes. Previously, **Tables S2 and S4** combined SNP-based gene annotations and MAGMA gene-based results in FUMA. For clarity, these analyses are now separated: SNP-based annotations in **S4, S14, and S36 Tables**, and MAGMA gene-level results in **S5, S15, and S37 Tables**.

Novelty was defined only at the locus level by: (1) genome-wide significance in our GWAS but not in comparison ancestries, (2) colocalization evidence for population-specific signals, and (3) absence of the nearest gene from SNP- and gene-based lists, yielding four novel loci for height and none for FSS. As MAGMA aggregates SNP associations to genes, a few variants can implicate multiple genes via positional, eQTL, or chromatin mapping. Because such signals reflect regulatory complexity rather than true novelty, we define novelty only at the locus level, not at the gene level. These revisions are highlighted in the **Results (page 7, lines 148–164; page 8, lines 182–191)** and **Materials and methods (page 18, lines 419–438)**.

10. Line 282 Please provide specific diagnosis code uses in this study.

We thank the reviewer for this helpful comment. In the CMUH cohort, disease phenotypes were defined using PheCODEs mapped from ICD-9-CM diagnoses [1], with follow-up from birth until the first PheCODE-defined disease or last data entry (**S24 and S26 Tables**). Cases were defined as ≥2 outpatient visits or one inpatient admission, and diseases with fewer than 200 cases were excluded. These revisions are highlighted in the **Materials and methods** (**page 17, lines 398–402**).

11. PheWAS does not only indicate phenome-wide screening of associations for PRS. PheWAS includes pleiotropic scan of specific variants or phenome wide GWAS. Using more specific wording will help readers.

We appreciate the reviewer’s clarification that PheWAS encompasses pleiotropic scans of specific variants and broad phenome-wide GWAS in addition to PRS-based analyses. To avoid ambiguity, we have revised the manuscript to consistently use the term PRS-PheWAS and explicitly state that our study applied PheWAS methods specifically to polygenic risk scores. We also added a clarifying note to emphasize that while PheWAS has broader applications, our analyses focused on PRS-PheWAS (**Results, page 9, lines 207-229)** (**Materials and methods, page 19, lines 461–464**).

12. Line 343 "We conducted genome-wide association study (GWAS) analyses to obtain body mass index (BMI) and body fat percentage GWAS summary statistics from the Taiwan Biobank database" Is this relevant to this study? I also did not find description about data availability of GWAS for Height and FSS conducted in this study.

We thank the reviewer for noting this error, which has been corrected to indicate that we conducted GWAS to obtain body height summary statistics from the Taiwan Biobank database. We have also revised the **Data Availability statement (page 21, lines 502–510)** to clarify the availability of GWAS data for height and FSS generated in this study.

**References:**

1.	Bastarache L. Using Phecodes for Research with the Electronic Health Record: From PheWAS to PheRS. Annu Rev Biomed Data Sci. 2021;4:1-19. Epub 2021/09/02. doi: 10.1146/annurev-biodatasci-122320-112352. PubMed PMID: 34465180; PubMed Central PMCID: PMCPMC9307256.

2.	Ge T, Chen CY, Ni Y, Feng YA, Smoller JW. Polygenic prediction via Bayesian regression and continuous shrinkage priors. Nat Commun. 2019;10(1):1776. Epub 2019/04/18. doi: 10.1038/s41467-019-09718-5. PubMed PMID: 30992449; PubMed Central PMCID: PMCPMC6467998.

3.	Farland LV, Missmer SA, Bijon A, Gusto G, Gelot A, Clavel-Chapelon F, et al. Associations among body size across the life course, adult height and endometriosis. Hum Reprod. 2017;32(8):1732-42. Epub 2017/06/08. doi: 10.1093/humrep/dex207. PubMed PMID: 28591798; PubMed Central PMCID: PMCPMC5850750.

4.	Parasar P, Ozcan P, Terry KL. Endometriosis: Epidemiology, Diagnosis and Clinical Management. Curr Obstet Gynecol Rep. 2017;6(1):34-41. Epub 2017/12/26. doi: 10.1007/s13669-017-0187-1. PubMed PMID: 29276652; PubMed Central PMCID: PMCPMC5737931.

5.	Aarestrup J, Jensen BW, Ulrich LG, Hartwell D, Trabert B, Baker JL. Birth weight, childhood body mass index and height and risks of endometriosis and adenomyosis. Ann Hum Biol. 2020;47(2):173-80. Epub 2020/03/11. doi: 10.1080/03014460.2020.1727011. PubMed PMID: 32151170; PubMed Central PMCID: PMCPMC7905846.

6.	Ahmed ML, Ong KK, Dunger DB. Childhood obesity and the timing of puberty. Trends Endocrinol Metab. 2009;20(5):237-42. Epub 2009/06/23. doi: 10.1016/j.tem.2009.02.004. PubMed PMID: 19541497.

7.	Sakaue S, Kanai M, Tanigawa Y, Karjalainen J, Kurki M, Koshiba S, et al. A cross-population atlas of genetic associations for 220 human phenotypes. Nat Genet. 2021;53(10):1415-24. Epub 2021/10/02. doi: 10.1038/s41588-021-00931-x. PubMed PMID: 34594039.

8.	Nam K, Kim J, Lee S. Genome-wide study on 72,298 individuals in Korean biobank data for 76 traits. Cell Genom. 2022;2(10):100189. Epub 2023/02/14. doi: 10.1016/j.xgen.2022.100189. PubMed PMID: 36777999; PubMed Central PMCID: PMCPMC9903843.

9.	Yengo L, Vedantam S, Marouli E, Sidorenko J, Bartell E, Sakaue S, et al. A saturated map of common genetic variants associated with human height. Nature. 2022;610(7933):704-12. Epub 2022/10/13. doi: 10.1038/s41586-022-05275-y. PubMed PMID: 36224396; PubMed Central PMCID: PMCPMC9605867 shareholder in Zealand Pharma A/S and Novo Nordisk A/S. G.C.-P. is an employee of 23andMe. M.E.K. is employed by SYNLAB Holding Deutschland GmbH. H.L.L. receives support from a consulting contract between Data Tecnica International and the National Institute on Aging (NIA), National Institutes of Health (NIH). As of January 2020, A. Mahajan is an employee of Genentech, and a holder of Roche stock. I.N. is an employee and stock owner of Gilead Sciences; this work was conducted before employment by Gilead Sciences. J. Shi is employed by and holds stock or stock options in 23andMe. C. Sidore is an employee of Regeneron. V. Steinthorsdottir is employed by deCODE Genetics/Amgen. Since completing the work contributed to this paper, D.J.T. has left the University of Cambridge and is now employed by Genomics PLC. G.T. is employed by deCODE Genetics/Amgen. S.W.v.d.L. has received Roche funding for unrelated work. H.B. has consultant arrangements with Chiesi Pharmaceuticals and Boehringer Ingelheim. M. J. Caulfield is Chief Scientist for Genomics England, a UK Government company. M. J. Cutler has served on the advisory board or consulted for Biosense Webster, Janssen Scientific Affairs and Johnson & Johnson. S.M.D. receives research support from RenalytixAI and personal consulting fees from Calico Labs, outside the scope of the current research. P.T.E. receives sponsored research support from Bayer AG and IBM Health, and he has served on advisory boards or consulted for Bayer AG, Quest Diagnostics, MyoKardia and Novartis. P. Kirchhof has received support from several drug and device companies active in atrial fibrillation, and has received honoraria from several such companies in the past, but not in the last three years. P. Kirchhof is listed as inventor on two patents held by University of Birmingham (Atrial Fibrillation Therapy WO 2015140571, Markers for Atrial Fibrillation WO 2016012783). G.D.K. has given talks, attended conferences and participated in trials sponsored by Amgen, MSD, Lilly, Vianex and Sanofi, and has also accepted travel support to conferences from Amgen, Sanofi, MSD and Elpen. S. A. Lubitz previously received sponsored research support from Bristol Myers Squibb, Pfizer, Bayer AG, Boehringer Ingelheim, Fitbit and IBM, and has consulted for Bristol Myers Squibb, Pfizer, Bayer AG and Blackstone Life Sciences. S. A. Lubitz is a current employee of Novartis Institute of Biomedical Research. W.M. reports grants and personal fees from AMGEN, BASF, Sanofi, Siemens Diagnostics, Aegerion Pharmaceuticals, Astrazeneca, Danone Research, Numares, Pfizer and Hoffmann LaRoche; personal fees from MSD and Alexion; and grants from Abbott Diagnostics, all outside the submitted work. W.M. is employed with Synlab Holding Deutschland. M.A.N. receives support from a consulting contract between Data Tecnica International and the National Institute on Aging (NIA), National Institutes of Health (NIH). S.N. is a scientific advisor to Circle software, ADAS software, CardioSolv and ImriCor and receives grant support from Biosense Webster, ADAS software and ImriCor. H. Schunkert has received honoraria for consulting from AstraZeneca, MSD, Merck, Daiichi, Servier, Amgen and Takeda Pharma. He has further received honoraria for lectures and/or chairs from AstraZeneca, BayerVital, BRAHMS, Daiichi, Medtronic, Novartis, Sanofi and Servier. P.S. has received research awards from Pfizer. The members of the 23andMe Research Team are employed by and hold stock or stock options in 23andMe. The views expressed in this article are those of the author(s) and not necessarily those of the NHS, the NIHR or the Department of Health. M. I. McCarthy has served on advisory panels for Pfizer, Novo Nordisk and Zoe Global, and has received honoraria from Merck, Pfizer, Novo Nordisk and Eli Lilly and research funding from Abbvie, AstraZeneca, Boehringer Ingelheim, Eli Lilly, Janssen, Merck, Novo Nordisk, Pfizer, Roche, Sanofi Aventis, Servier and Takeda. As of June 2019, M. I. McCarthy is an employee of Genentech, and a holder of Roche stock. C.J.O. is a current employee of Novartis Institute of Biomedical Research. U.T. is employed by deCODE Genetics (Amgen). K.S. is employed by deCODE Genetics (Amgen). A. Auton is employed by and holds stock or stock options in 23andMe. G.R.A. is an employee of Regeneron Pharmaceuticals and owns stock and stock options for Regeneron Pharmaceuticals. C.J.W.'s spouse is employed by Regeneron. A.E.L. is currently employed by and holds stock in Regeneron Pharmaceuticals. J.N.H. holds equity in Camp4 Therapeutics. The remaining authors declare no competing interests.

10.	Giambartolomei C, Vukcevic D, Schadt EE, Franke L, Hingorani AD, Wallace C, et al. Bayesian test for colocalisation between pairs of genetic association studies using summary statistics. PLoS Genet. 2014;10(5):e1004383. Epub 2014/05/17. doi: 10.1371/journal.pgen.1004383. PubMed PMID: 24830394; PubMed Central PMCID: PMCPMC4022491.

11.	Browning BL, Zhou Y, Browning SR. A One-Penny Imputed Genome from Next-Generation Reference Panels. Am J Hum Genet. 2018;103(3):338-48. Epub 2018/08/14. doi: 10.1016/j.ajhg.2018.07.015. PubMed PMID: 30100085; PubMed Central PMCID: PMCPMC6128308.

12.	Liu TY, Lin CF, Wu HT, Wu YL, Chen YC, Liao CC, et al. Comparison of multiple imputation algorithms and verification using whole-genome sequencing in the CMUH genetic biobank. Biomedicine (Taipei). 2021;11(4):57-65. Epub 2022/03/01. doi: 10.37796/2211-8039.1302. PubMed PMID: 35223420; PubMed Central PMCID: PMCPMC8823485.
