# Supplementary material for: Unraveling the genetic links between stature and disease in East Asians: A multi-biobank genetic correlation and risk prediction study
Source: PLoS Genet. 2026 Mar 13;22(3):e1012030. doi: 10.1371/journal.pgen.1012030 (PMC13001983; doi:10.1371/journal.pgen.1012030)
Supplement: Attachment — Submitted filename: Response_to_PLOS genetics_12232025.docx [file pgen.1012030.s061.docx]

PLOS Genetics

MS ID: PGENETICS-D-25-00676R1

MS TITLE: Unraveling the genetic links between stature and disease in East Asians: A multi-biobank genetic correlation and risk prediction study

Dear Professors Themistocles L. Assimes, Anne O'Donnell-Luria, Aimée Dudley, and Anne Goriely:

Thank you for your e-mail dated November 17, informing us that you and the reviewer have suggested a revision for our manuscript. We greatly appreciate this opportunity, and we have made changes with red marks as per the reviewers’ comments. Our point-by-point responses to the reviewers’ comments are given below.

We believe that the revision has substantially improved our manuscript and hope that the revised version is now suitable for further review.

Thank you for considering our work. We eagerly await your response.

Sincerely,

Fuu-Jen Tsai

China Medical University

No. 2, Yuh-Der Road, Taichung, Taiwan 40442

+886 4 22062121

[000704@tool.caaumed.org.tw](mailto:000704@tool.caaumed.org.tw)

**Responses to comments by Reviewer 1:**

Lin et al. have submitted a revised version of their manuscript, with several additional analyses added. Some of my previous comments have been addressed but not all and the new additions have generated more concerns for me.

One major comment is that currently the supplementary tables in word are impossible to consider in a meaningful way. The authors should provide all supplementary tables in an excel format.

We appreciate the reviewer’s feedback regarding the accessibility of the data. Accordingly, we converted all supplementary tables into Excel (.xlsx) files to allow for meaningful review and analysis.

**In addition:**

1. The authors have removed the sentence “Herein, GWAS, PheWAS, and PRS survival analyses were performed, revealing that genetically taller stature is linked to increased risks of AF and endometriosis, whereas shorter stature is associated with later onset and a reduced risk of endometriosis individuals”, since they age of onset was not properly tested. But they still mention in the abstract and the discussion that "Higher height PRS further predicted an earlier and higher AF incidence, consistently replicated in survival analyses of meta-analysis–derived PRSs". It is not clear from their analysis, how the "earlier AF incidence" is justified.

We thank the reviewer for this important distinction. We agree that our previous phrasing regarding "earlier incidence" could be misinterpreted as a direct measurement of individual age of onset. We revised the text in the manuscript to clarify that our findings refer to the earlier divergence of cumulative incidence curves based on Kaplan–Meier and Cox proportional hazard analyses, rather than a direct test of onset age.

To justify this, we highlight that the cumulative incidence curves for the high PRS group (80–100%) and low PRS group (0–20%) diverged significantly (log-rank *P* = 5.48 × 10⁻¹¹) in the CMUH cohort. Specifically, the high-risk group reached a 10% cumulative incidence threshold at age 78.2, compared to age 81.6 for the low-risk group. This 3.4-year difference demonstrates a significantly steeper risk trajectory and earlier accumulation of cases in the high-PRS group.

We updated the manuscript to reflect this precise terminology in the Abstract, Author Summary, Results, and Discussion (**Abstract, page 3, lines 65–67**) (**Author summary, page 4, lines 85–86**) (**Results, page 13, lines 299–306**) (**Discussion, pages 13–14, lines 317–322; page 15, lines 368–369; page 16, lines 393–394**).

2. The locus definition is still unclear to me; there is no description as to what a "distinct" locus means in terms of distance.

We thank the reviewer for requesting clarification on this important technical detail. In the revised manuscript, we explicitly defined the criteria for a "distinct locus." We employed the default settings of LocusZoom (locuszoom.org), defining a locus as the genomic region within ±500 kb of a genome-wide significant lead SNP, including all variants in linkage disequilibrium (LD) at r² ≥ 0.4 based on the 1000 Genomes Phase 3 East Asian reference panel. To ensure independence, loci with overlapping intervals were merged, and only non-overlapping genomic regions were considered ‘distinct loci’. We updated the Methods section to include this detailed definition (**Materials and methods, page 19, lines 450–455**).

3. The classification of novel vs known loci is very confusing. Standard practice is to check all published variants for the trait of interest (from the GWAS catalog) and check via LD if current variants are proxies or not of published variants. I am unsure as to why the authors have performed a co-localization analysis, as this is more suitable for identifying variants that affect more than one traits. If they wish to identify independent/novel variants with published loci, them they need to perform conditional analysis not co-localization.

We thank the reviewer for this constructive feedback and agree that distinguishing novel loci requires rigorous conditional analysis and linkage disequilibrium (LD) profiling rather than colocalization. Consequently, we revised our approach to implement a stringent three-stage verification framework.

We first compiled a comprehensive reference catalog of established height-associated variants from the GWAS Catalog (https://www.ebi.ac.uk/gwas/efotraits/OBA_VT0001253) and major multi-ancestry meta-analyses [1-3]. Next, we assessed internal and external independence using GCTA-COJO joint and conditional analyses (https://github.com/kheilbron/cojo_pipe) [4], prioritizing signals that retained genome-wide significance (*P*_cond_ < 5 × 10^-8^) after adjusting for reference variants. Finally, to strictly exclude proxies, we defined loci as novel only if they exhibited negligible linkage disequilibrium (r^2^ < 0.1) with known signals.

Using this framework, we identified 16 novel loci. We clarify that colocalization analysis is now retained solely to assess the consistency of association signals across different ancestries (e.g., between TWB and other East Asian or European cohorts) rather than to define novelty in the revised manuscript. This improved classification strategy is detailed in the **Methods section (Page 20, lines 470–488**) and **Supplementary Tables (S3 and S14 Tables)**.

4. Lines 173-176 are more suitable in the Methods section.

We thank the reviewer for this helpful suggestion regarding manuscript organization. We agree that the details originally presented in Lines 173–176 differ from the primary results and are more appropriate for the methodological description. Accordingly, we moved this text to the **Materials and Methods section** in the revised manuscript (P**ages 17–18, lines 417–420**).

5. Based on the FSS description, it becomes apparent that only children are included in the cases (since the ICD codes only refer the children conditions), while all the controls are adults. This raises major concerns about the suitability of the control group and the selection bias introduced here.

We thank the reviewer for highlighting this critical point regarding study design and potential selection bias. We acknowledge the age discrepancy between pediatric FSS cases and adult controls and provide the following justification: we selected adult controls (>18 years) with finalized height ≥75th percentile to maximize phenotypic certainty. Unlike pediatric controls, whose growth trajectories are ongoing, adults achieve their final stature, thereby eliminating the risk of misclassifying individuals who might later manifest short stature. To statistically account for the age difference, all association models (conducted via REGENIE) [5] were adjusted for age, sex, and the first 10 principal components using a case–control framework.

Crucially, to empirically address the concern regarding selection bias, we performed a sensitivity analysis using an alternative control set explicitly matched to cases by age and sex. The GWAS signals derived from this age-matched analysis were highly concordant with our primary analysis, confirming that our findings are robust to the control definition and are not driven by age imbalance. We have added a detailed explanation of the control selection rationale to the **Methods (Pages 17–18, lines 419–427)** and included the results of the sensitivity analysis in the **Results section (Page 9, lines 206–209)**, supported by **Supplementary Figures S8 and S9 and Supplementary Table S12**.

6. It is unclear if the FUMA and MAGMA analysis are meant to identify causative genes but seems counter-intuitive that 5 variants are mapped to 27 genes with no prioritization.

We thank the reviewer for this constructive comment and apologize for the lack of clarity regarding our gene mapping strategy. We agree that listing 27 genes for five loci requires distinct prioritization. The initial list represents a comprehensive candidate set derived from broad functional annotation rather than definitive causal assignment. To address this issue, we clarified our tiered prioritization framework in the revised manuscript. First, FUMA SNP2GENE [6, 7] mapped variants to 26 biologically plausible genes, with DCAF16, NCAPG, and LCORL showing the strongest SNP-based evidence (minGWAS *P* ≤ 3.31 × 10⁻¹³). Second, MAGMA gene-based analysis [6, 7] narrowed the focus to 8 significant genes, specifically prioritizing *IGF1*, *ATF7*, and *CCDC53* (**S17 Table**). Finally, we performed Ingenuity Pathway Analysis (IPA) to further prioritize candidates enriched in critical growth pathways, identifying 'Cell Cycle: G1/S Checkpoint Regulation' and 'Growth Hormone Signaling' as key biological drivers (**S18 and S19 Tables**) (**S6 and S7 Figs**). We revised the **Results section** to explicitly present this prioritization hierarchy (**Page 9, lines 201–204**).

7. Line 195 implies causality that is not supported by the type of the analyses presented in that section. Same for line204.

We thank the reviewer for this accurate observation. We agree that the original wording in lines 195 and 204 inadvertently implied causality, which is not supported by the cross-trait genetic correlation and hierarchical clustering analyses presented in that section. These methods identify shared genetic architecture rather than directional causal effects. Accordingly, we have revised the text to strictly employ non-causal terminology (e.g., "associated with" or "shared genetic signals") to accurately reflect the findings’ associative nature. These revisions can be found in the **Results section** (**Page 9, lines 213–214**; **Pages 9–10, lines 221–224**).

8. PheWAS results need to be corrected for multiple testing (see line 220).

We thank the reviewer for this important reminder to ensure statistical rigor. We have applied a strict Bonferroni correction across all 1,090 tested phenotypes (*P* < 0.05/1,090) to adjust for multiple testing. In the revised manuscript, we explicitly report only those associations that retained significance after this adjustment. Under this stringent threshold, the PheWAS of height polygenic risk scores identified 13 significant disease phenotypes, with the strongest associations observed for 'lack of normal physiological development', 'short stature', atrial fibrillation (AF), and endometriosis. We updated the **Results section (Page 10, line 239)** and **Supplementary Table S28** to reflect these corrected findings.

9. Line 223 mentions an "independent" cohort but there is no information of that cohort anywhere.

We thank the reviewer for highlighting this omission. In the revised manuscript, we clarified that the "independent cohort" refers to a validation dataset specifically constructed for the PRS-PheWAS analysis. This cohort was derived from the CMUH_410K_SNP dataset linked to electronic health records and was rigorously filtered to ensure no overlap with the FSS GWAS discovery samples. Specifically, after excluding all individuals used in the prior GWAS, removing duplicates, and filtering for data quality, we retained 258,931 participants (mean age 47.1 years; mean follow-up 46.9 years). We have added a comprehensive description of this cohort's construction, inclusion criteria, and demographics to the **Results section (Pages 10–11, lines 241–247)**, supported by **Supplementary Figure S11 and Supplementary Table S29**.

10. There seems to be lack of independent studies for validating the PRSs. The same cohort is being used to derive GWAS effect sizes, train, test and validate the PRS. This will overestimate the PRS performance.

We thank the reviewer for raising this critical point regarding the risk of overfitting. We fully agree that overlapping discovery and target samples can inflate PRS performance estimates. To avoid this, our study design prioritized strict sample separation and external replication. First, we validated our findings in the Biobank Japan (BBJ) cohort, entirely independent of the CMUH discovery dataset. Second, within the CMUH biobank, we constructed a specific validation cohort for the FSS PRS–PheWAS by rigorously excluding all individuals used in the discovery GWAS, as well as duplicates and those failing quality control (N = 258,931). Using these independent target datasets, we observed consistent associations: higher height PRSs were significantly associated with increased atrial fibrillation risk in both the external BBJ cohort (OR = 1.14–1.15, *P* < 2.0×10⁻¹⁶) and the independent CMUH validation set (OR = 1.12, *P* = 3.0×10⁻¹⁸).

Furthermore, the association with endometriosis was significant in the independent CMUH set (OR = 1.07, *P* = 4.8×10⁻⁹), though it did not reach significance in BBJ. These replication results across independent datasets support the robustness of our PRS performance and confirm that our findings are not driven by sample overlap. We have detailed this validation strategy in the **Results section (Pages 10–11, lines 241-257)** and **Supplementary Figure S11, Table S29, and Table S31**.

11. The authors seem to use an AF PRS and an endometriosis PRS but there is no information on how these PRS were derived.

We thank the reviewer for noting this omission. We apologize for not explicitly detailing the derivation of the disease-specific polygenic risk scores (PRSs) used as covariates. Both scores were generated using the same PRS-CS pipeline as in our main analyses. Specifically, AF and endometriosis PRSs were computed for each CMUH participant using Biobank Japan (BBJ) GWAS summary statistics. We applied the PRS-CS Bayesian regression method with a fixed global shrinkage parameter (ϕ = 1 × 10⁻²) and the 1000 Genomes Phase 3 East Asian LD reference panel. The resulting posterior SNP effect sizes were used to calculate scores, which were then standardized per SD for association testing. We have added this information to **S32 Table**.

12. The authors have performed a series of MR analysis but there is no description of this in the methods. In addition, it would be helpful if the results presented in figure S8 were also included in a supplementary table.

We thank the reviewer for this constructive suggestion. We have incorporated a comprehensive description of our Mendelian randomization (MR) framework into the **Materials and Methods section (Page 22, lines 520–533)**.

This revision details our rigorous instrument selection criteria—utilizing genome-wide significant (*P* < 5 × 10⁻⁸), linkage disequilibrium-independent (r² < 0.001), and minor allele frequency-filtered (MAF > 0.01) variants with strong F-statistics (> 10), and outlines the application of univariable (IVW, MR-Egger, MR-PRESSO) and multivariable sensitivity analyses.

Regarding the presentation of results, we confirm that the complete numerical data underlying **the updated Supplementary Figure S12** are provided in **Supplementary Tables S34 and S35** (reporting univariable and multivariable estimates adjusted for age at menarche and body weight) and **Supplementary Table S33** (listing the instrumental variables). We have revised the text to explicitly direct readers to these tables (**Results, page 12, lines 273–279**).

**Responses to comments by Reviewer 2:**

The authors have substantially improved their manuscript and addressed all of my concerns.

We sincerely thank the reviewer for their positive evaluation and for acknowledging that the revisions have addressed their concern. We appreciate the reviewer’s careful assessment, which has helped us strengthen the manuscript.

**Responses to comments by Reviewer 3:**

Lin and colleagues have significantly improved the overall transparency of their work; the readability has also been significantly improved. I am happy with authors’ response regarding points I previously raised.

We thank the reviewer for their careful re-evaluation and for encouraging feedback. We are pleased that the revisions have improved the transparency and readability of the manuscript, and we appreciate the reviewer’s acknowledgment that the concerns raised in the previous round have been satisfactorily addressed.

1. One major point I find is that, I can see that the authors had added the part using Mendelian randomization to examine potential causal associations, but I did not find the detailed methodology for MR in the Methods section. Particularly, how instruments were selected in both univariable MR and multivariable MR; if the three core assumptions for MR were tested (I can see that the authors have adopted MR-PRESSO, but only from the supplementary materials); and which R package was used for these analyses. MVMR estimates could be biased if the conditional F-statistics are low (instrument strength). These details should be provided.

We thank the reviewer for this constructive suggestion and have incorporated a comprehensive description of our Mendelian randomization (MR) framework into the **Materials and Methods section (Page 22, lines 520–533)**. This revision details our rigorous instrument selection criteria—utilizing genome-wide significant (*P* < 5 × 10⁻⁸), linkage disequilibrium-independent (r² < 0.001), and MAF-filtered (> 0.01) variants with strong F-statistics (> 10) —and outlines the application of univariable (IVW, MR-Egger, MR-PRESSO) and multivariable sensitivity analyses using the TwoSampleMR R package.

Regarding the presentation of results, we confirm that the complete numerical data underlying **the updated Supplementary Figure S12** are provided in **Supplementary Tables S34 and S35** (reporting univariable and multivariable estimates adjusted for age at menarche and body weight) and **Supplementary Table S33** (listing the instrumental variables). We have revised the text to explicitly direct readers to these tables (**Results, page 12, lines 273–279**).

**Minor point:**

1. I was trying to investigate some of the supplementary tables, but tables in a word document make them very difficult to navigate, compared to a spreadsheet file like excel. I’m not sure if this is the requirement from the journal.

We thank the reviewer for this helpful suggestion. To improve accessibility while meeting journal formatting requirements, we have provided the supplementary tables in the Excel format in the revised submission.

**Responses to comments by Reviewer 4:**

Thank you for the comprehensive revisions to the manuscript and the inclusion of additional interestingfindings. I have no further comments.

We sincerely thank the reviewer for their careful re-evaluation and for the positive feedback. We are glad that the revisions and additional findings addressed the reviewer’s concerns, and we appreciate their time and constructive input throughout the review process.

**References:**

1.	Yengo L, Vedantam S, Marouli E, Sidorenko J, Bartell E, Sakaue S, et al. A saturated map of common genetic variants associated with human height. Nature. 2022;610(7933):704-12. Epub 2022/10/13. doi: 10.1038/s41586-022-05275-y. PubMed PMID: 36224396; PubMed Central PMCID: PMCPMC9605867 shareholder in Zealand Pharma A/S and Novo Nordisk A/S. G.C.-P. is an employee of 23andMe. M.E.K. is employed by SYNLAB Holding Deutschland GmbH. H.L.L. receives support from a consulting contract between Data Tecnica International and the National Institute on Aging (NIA), National Institutes of Health (NIH). As of January 2020, A. Mahajan is an employee of Genentech, and a holder of Roche stock. I.N. is an employee and stock owner of Gilead Sciences; this work was conducted before employment by Gilead Sciences. J. Shi is employed by and holds stock or stock options in 23andMe. C. Sidore is an employee of Regeneron. V. Steinthorsdottir is employed by deCODE Genetics/Amgen. Since completing the work contributed to this paper, D.J.T. has left the University of Cambridge and is now employed by Genomics PLC. G.T. is employed by deCODE Genetics/Amgen. S.W.v.d.L. has received Roche funding for unrelated work. H.B. has consultant arrangements with Chiesi Pharmaceuticals and Boehringer Ingelheim. M. J. Caulfield is Chief Scientist for Genomics England, a UK Government company. M. J. Cutler has served on the advisory board or consulted for Biosense Webster, Janssen Scientific Affairs and Johnson & Johnson. S.M.D. receives research support from RenalytixAI and personal consulting fees from Calico Labs, outside the scope of the current research. P.T.E. receives sponsored research support from Bayer AG and IBM Health, and he has served on advisory boards or consulted for Bayer AG, Quest Diagnostics, MyoKardia and Novartis. P. Kirchhof has received support from several drug and device companies active in atrial fibrillation, and has received honoraria from several such companies in the past, but not in the last three years. P. Kirchhof is listed as inventor on two patents held by University of Birmingham (Atrial Fibrillation Therapy WO 2015140571, Markers for Atrial Fibrillation WO 2016012783). G.D.K. has given talks, attended conferences and participated in trials sponsored by Amgen, MSD, Lilly, Vianex and Sanofi, and has also accepted travel support to conferences from Amgen, Sanofi, MSD and Elpen. S. A. Lubitz previously received sponsored research support from Bristol Myers Squibb, Pfizer, Bayer AG, Boehringer Ingelheim, Fitbit and IBM, and has consulted for Bristol Myers Squibb, Pfizer, Bayer AG and Blackstone Life Sciences. S. A. Lubitz is a current employee of Novartis Institute of Biomedical Research. W.M. reports grants and personal fees from AMGEN, BASF, Sanofi, Siemens Diagnostics, Aegerion Pharmaceuticals, Astrazeneca, Danone Research, Numares, Pfizer and Hoffmann LaRoche; personal fees from MSD and Alexion; and grants from Abbott Diagnostics, all outside the submitted work. W.M. is employed with Synlab Holding Deutschland. M.A.N. receives support from a consulting contract between Data Tecnica International and the National Institute on Aging (NIA), National Institutes of Health (NIH). S.N. is a scientific advisor to Circle software, ADAS software, CardioSolv and ImriCor and receives grant support from Biosense Webster, ADAS software and ImriCor. H. Schunkert has received honoraria for consulting from AstraZeneca, MSD, Merck, Daiichi, Servier, Amgen and Takeda Pharma. He has further received honoraria for lectures and/or chairs from AstraZeneca, BayerVital, BRAHMS, Daiichi, Medtronic, Novartis, Sanofi and Servier. P.S. has received research awards from Pfizer. The members of the 23andMe Research Team are employed by and hold stock or stock options in 23andMe. The views expressed in this article are those of the author(s) and not necessarily those of the NHS, the NIHR or the Department of Health. M. I. McCarthy has served on advisory panels for Pfizer, Novo Nordisk and Zoe Global, and has received honoraria from Merck, Pfizer, Novo Nordisk and Eli Lilly and research funding from Abbvie, AstraZeneca, Boehringer Ingelheim, Eli Lilly, Janssen, Merck, Novo Nordisk, Pfizer, Roche, Sanofi Aventis, Servier and Takeda. As of June 2019, M. I. McCarthy is an employee of Genentech, and a holder of Roche stock. C.J.O. is a current employee of Novartis Institute of Biomedical Research. U.T. is employed by deCODE Genetics (Amgen). K.S. is employed by deCODE Genetics (Amgen). A. Auton is employed by and holds stock or stock options in 23andMe. G.R.A. is an employee of Regeneron Pharmaceuticals and owns stock and stock options for Regeneron Pharmaceuticals. C.J.W.'s spouse is employed by Regeneron. A.E.L. is currently employed by and holds stock in Regeneron Pharmaceuticals. J.N.H. holds equity in Camp4 Therapeutics. The remaining authors declare no competing interests.

2.	Sakaue S, Kanai M, Tanigawa Y, Karjalainen J, Kurki M, Koshiba S, et al. A cross-population atlas of genetic associations for 220 human phenotypes. Nat Genet. 2021;53(10):1415-24. Epub 2021/10/02. doi: 10.1038/s41588-021-00931-x. PubMed PMID: 34594039.

3.	Nam K, Kim J, Lee S. Genome-wide study on 72,298 individuals in Korean biobank data for 76 traits. Cell Genom. 2022;2(10):100189. Epub 2023/02/14. doi: 10.1016/j.xgen.2022.100189. PubMed PMID: 36777999; PubMed Central PMCID: PMCPMC9903843.

4.	Yang J, Ferreira T, Morris AP, Medland SE, Genetic Investigation of ATC, Replication DIG, et al. Conditional and joint multiple-SNP analysis of GWAS summary statistics identifies additional variants influencing complex traits. Nat Genet. 2012;44(4):369-75, S1-3. Epub 2012/03/20. doi: 10.1038/ng.2213. PubMed PMID: 22426310; PubMed Central PMCID: PMCPMC3593158.

5.	Mbatchou J, Barnard L, Backman J, Marcketta A, Kosmicki JA, Ziyatdinov A, et al. Computationally efficient whole-genome regression for quantitative and binary traits. Nat Genet. 2021;53(7):1097-103. Epub 2021/05/22. doi: 10.1038/s41588-021-00870-7. PubMed PMID: 34017140.

6.	de Leeuw CA, Mooij JM, Heskes T, Posthuma D. MAGMA: generalized gene-set analysis of GWAS data. PLoS Comput Biol. 2015;11(4):e1004219. Epub 2015/04/18. doi: 10.1371/journal.pcbi.1004219. PubMed PMID: 25885710; PubMed Central PMCID: PMCPMC4401657.

7.	Watanabe K, Taskesen E, van Bochoven A, Posthuma D. Functional mapping and annotation of genetic associations with FUMA. Nat Commun. 2017;8(1):1826. Epub 2017/12/01. doi: 10.1038/s41467-017-01261-5. PubMed PMID: 29184056; PubMed Central PMCID: PMCPMC5705698.
